# A Proposal for a Coordinated Effort for the Determination of Brainwide Neuroanatomical Connectivity in Model Organisms at a Mesoscopic Scale

**DOI:** 10.1371/journal.pcbi.1000334

**Published:** 2009-03-27

**Authors:** Jason W. Bohland, Caizhi Wu, Helen Barbas, Hemant Bokil, Mihail Bota, Hans C. Breiter, Hollis T. Cline, John C. Doyle, Peter J. Freed, Ralph J. Greenspan, Suzanne N. Haber, Michael Hawrylycz, Daniel G. Herrera, Claus C. Hilgetag, Z. Josh Huang, Allan Jones, Edward G. Jones, Harvey J. Karten, David Kleinfeld, Rolf Kötter, Henry A. Lester, John M. Lin, Brett D. Mensh, Shawn Mikula, Jaak Panksepp, Joseph L. Price, Joseph Safdieh, Clifford B. Saper, Nicholas D. Schiff, Jeremy D. Schmahmann, Bruce W. Stillman, Karel Svoboda, Larry W. Swanson, Arthur W. Toga, David C. Van Essen, James D. Watson, Partha P. Mitra

**Affiliations:** 1 Cold Spring Harbor Laboratory, Cold Spring Harbor, New York, United States of America; 2 Department of Health Sciences, Boston University, Boston, Massachusetts, United States of America; 3 Department of Biological Sciences, University of Southern California, Los Angeles, California, United States of America; 4 Department of Radiology, Massachusetts General Hospital, Charlestown, Massachusetts, United States of America; 5 Department of Electrical Engineering, California Institute of Technology, Pasadena, California, United States of America; 6 New York State Psychiatric Institute, Columbia University Medical Center, New York, New York, United States of America; 7 The Neurosciences Institute, San Diego, California, United States of America; 8 Department of Pharmacology & Physiology, University of Rochester Medical Center, Rochester, New York, United States of America; 9 Allen Institute for Brain Science, Seattle, Washington, United States of America; 10 Department of Psychiatry, Brigham and Women's Hospital, Harvard Medical School, Boston, Massachusetts, United States of America; 11 School of Engineering and Science, Jacobs University Bremen, Bremen, Germany; 12 Center for Neuroscience, University of California Davis, Davis, California, United States of America; 13 Department of Neurosciences, University of California San Diego School of Medicine, La Jolla, California, United States of America; 14 Department of Physics, University of California San Diego, La Jolla, California, United States of America; 15 Donders Institute for Brain, Cognition, and Behaviour, Department of Cognitive Neuroscience, NeuroPI, Radboud University Nijmegen Medical Centre, Nijmegen, The Netherlands; 16 Department of Biology, California Institute of Technology, Pasadena, California, United States of America; 17 Department of Psychiatry, Columbia University Medical Center, New York, New York, United States of America; 18 College of Veterinary Medicine, Washington State University, Pullman, Washington, United States of America; 19 Department of Anatomy & Neurobiology, Washington University School of Medicine, St. Louis, Missouri, United States of America; 20 Department of Neurology, Weill Cornell Medical College, New York, New York, United States of America; 21 Department of Neurology, Beth Israel Deaconess Medical Center, Boston, Massachusetts, United States of America; 22 Department of Neurology, Massachusetts General Hospital, Boston, Massachusetts, United States of America; 23 Janelia Farm Research Campus, Howard Hughes Medical Institute, Ashburn, Virginia, United States of America; 24 Laboratory of NeuroImaging, Department of Neurology, University of California Los Angeles School of Medicine, Los Angeles, California, United States of America; Indiana University, United States of America

## Abstract

In this era of complete genomes, our knowledge of neuroanatomical circuitry remains surprisingly sparse. Such knowledge is critical, however, for both basic and clinical research into brain function. Here we advocate for a concerted effort to fill this gap, through systematic, experimental mapping of neural circuits at a mesoscopic scale of resolution suitable for comprehensive, brainwide coverage, using injections of tracers or viral vectors. We detail the scientific and medical rationale and briefly review existing knowledge and experimental techniques. We define a set of desiderata, including brainwide coverage; validated and extensible experimental techniques suitable for standardization and automation; centralized, open-access data repository; compatibility with existing resources; and tractability with current informatics technology. We discuss a hypothetical but tractable plan for mouse, additional efforts for the macaque, and technique development for human. We estimate that the mouse connectivity project could be completed within five years with a comparatively modest budget.

## Introduction

**Figure 1:**
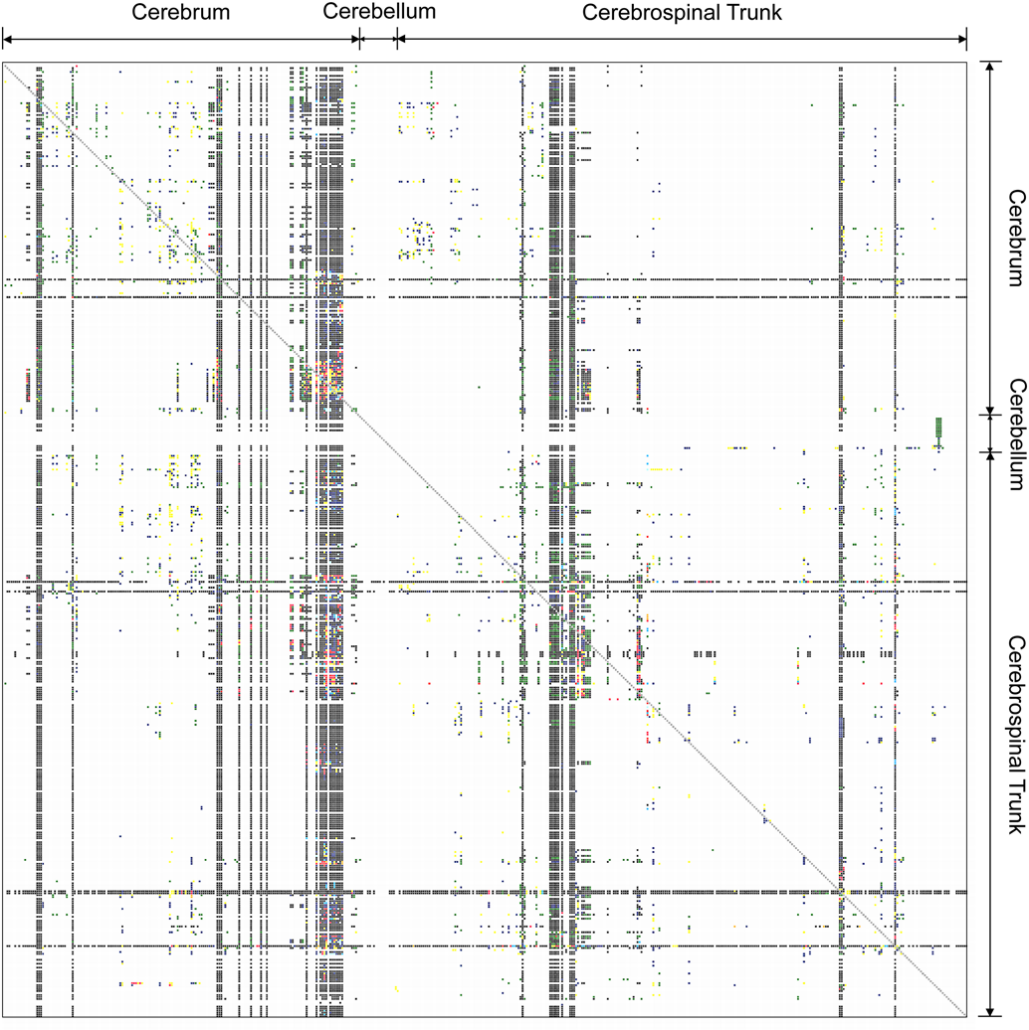
Current knowledge of rat brain connectivity available in the Brain Architecture Management System [2],[26]. This matrix shows information that has thus far been curated about projections between 486 discrete brain regions in the rat brain. Non-white entries indicate connections for which data are available. Black entries indicate the absence of a connection, and colored entries indicate reported connections of varying strength. The overall sparsity of this matrix (10.45% filled) is reflective of our lack of a unified understanding of brain connectivity in model organisms.

The defining architectural feature of the nervous system is that it forms a circuit. Unlike other tissues or organs, it is the patterns of axonal connections between neurons that determine the functioning of the brain. Nevertheless, more than a decade after Francis Crick and Ted Jones bemoaned the “Backwardness of Human Neuroanatomy [1],” our empirical knowledge about neuroanatomical connectivity in model organisms, including the mammalian species most widely used in biomedical research, remains surprisingly sparse. Efforts to manually curate neuroanatomical knowledge from the literature currently provide information about the reported presence or absence of ∼10% of all possible long-range projections between the roughly 500 identified brain regions in the rat [2] (Figure 1). While this number does not represent a comprehensive survey of the literature, it is clear that many possible projections have not yet been studied using modern tracing methods. In addition, the standard level of data analysis and presentation provides only a qualitative view of the known projections. Such paucity of empirical knowledge stands in contrast to the complete genomes now available for many organisms.

Here we argue the case for a coordinated effort across the neuroscience community to comprehensively determine neuroanatomical connectivity at a brainwide level in model organisms including the mouse, macaque, and eventually human. We discuss the important issues of resolution and rationale and survey the state of current knowledge and available techniques, then offer a basic outline for an experimental program and associated informatics requirements. The Allen Brain Atlas (ABA) for gene expression in the mouse [3] has demonstrated both the power of scaling up standardized techniques in neuroanatomical research and the feasibility of brainwide approaches. Numerous followup efforts to genome projects are also under way at various levels leading up to the phenotype. Time is therefore ripe for brainwide connectivity projects, to modernize neuroanatomical research, and to fill perhaps the largest lacuna in our knowledge about nervous system structure. The purpose of this article, which has resulted from discussions with a large and varied working group of experts, is to provide motivation and background for readers interested in brainwide connectivity projects, estimate resource requirements by analyzing a feasible scenario, recommend directions for such projects, and provide a platform for further discussions. The issues discussed here are likely to be relevant in implementing such a project through any combination of centralized and distributed efforts.

## The Mesoscopic Level of Resolution

It is clear that there exists some degree of nonrandom organization of the interconnections in the nervous system at multiple scales, including individual neurons, groups of neurons, architectonic regions and subcortical nuclei, and functional systems. *Macroscopic* brain organization, at the level of entire structural–functional systems and major fiber bundles, is somewhat understood but provides an insufficient description of the overall architecture. However, for complex vertebrate brains it is not currently technologically feasible to determine brainwide connectivity at the level of individual synapses. Further, while a statistical description is possible at this *microscopic* resolution, correspondence cannot be expected between individual brains described at the level of all synapses of all neurons. Significantly more invariance can be expected at a *mesoscopic* level where co-localized groups of neurons, perhaps of the same type or sharing common organizational features, are considered together as a unit, and projection patterns from these neuronal groups are studied over macroscopic distances. This level of connectivity is well-suited to aid our understanding of specific mental functions. A comprehensive mesoscopic wiring diagram, if available, would supply a meaningful skeleton that can be further augmented by the statistical characterization of microcircuitry at a finer scale (e.g., single neurons or cortical columns).

The existence and nature of invariant connectivity patterns across individual brains is itself a topic of research that can be addressed within a large-scale connectivity project. There is adequate evidence for mesoscopic architectural invariance in the form of cyto-, chemo-, and myelo-architectonically defined brain regions and from spatial gene expression patterns to proceed. In addition, however, a brainwide project executed with calculated redundancy will make it possible to empirically define the extent of such invariance. Further, if input and output connections are methodically determined along an appropriate anatomical grid, it should be possible to delineate the mesoscopic projection patterns in brain space without imposing a system of discrete anatomical parcels defined a priori.

## Scientific Rationale

The availability of mesoscopic circuit diagrams for model nervous systems would impact neuroscience research at nearly all levels. Because connectivity underlies nervous system function, any lack of such knowledge impedes the achievement of comprehensive understanding, even if complete information was present about cytoarchitecture, neuronal cell types, gene expression profiles, or other structural considerations. Furthermore, the connectional architecture of the nervous system—the *connectivity phenotype*—is a critical missing *link between genotype and behavioral phenotype*; the simultaneous availability of comprehensive genomic and neuroanatomical information will greatly narrow this gap. The scientific rationale can be further sharpened by examining the role of circuitry in experimental and theoretical approaches to the nervous system.

*Experimental design* in electrophysiological studies can be improved by explicit consideration of connectivity. For example, without any reference to underlying connectivity it is difficult to interpret measured physiological activity or the effects of microstimulation. Studies that consider the internal dynamics of the brain, including studies of selective attention, often make arguments about top-down or bottom-up processes, which are ultimately contingent on neuroanatomical information that is frequently deficient. Likewise, the lack of *empirical constraints on neural network models* remains an Achilles heel of that subject area, and such theoretical research would benefit greatly from added knowledge of connectional brain architecture.

Many *comparative and evolutionary studies* have also suffered from a phrenological emphasis on changes in morphological characteristics and relative sizes of parts of the nervous system, with less consideration of connectivity. Knowledge of the mesoscopic circuit diagrams for multiple model organisms will greatly advance comparative and evolutionary neuroanatomy, as has been the case for comparative and evolutionary genomics. This is highlighted by recent advances in understanding the relation between avian and mammalian brains. Purely structural considerations, such as the presence of a layered cortex in mammals, had led to incorrect homological identification of avian telencephalic structures with mammalian basal ganglia. Connectivity considerations have led to a profound revision of this view, leading to a new nomenclature for avian brain compartments [4].

## Biomedical Rationale

Neurological and neuropsychiatric disorders are responsible for approximately 30% of the total burden of illness in the United States, according to the World Health Organization's estimated Disability Adjusted Life Years (DALYs) for 2002 (http://www.who.int/healthinfo/global_burden_disease/en/index.html). The dominant paradigms for understanding such disorders have involved focal lesions, widespread neurodegeneration, vascular compromise, and neurotransmitter dysregulation, with circuit considerations playing a comparatively minor role. It has long been known, however, that disruptions in neural connectivity can underlie human brain disease [5],[6]. In disorders with no identified genetic component (e.g., traumatic brain injury or infectious disease), dysfunction arises directly from a disruption of the normal circuit. For those with heritable susceptibility effects, genetic polymorphism and cellular processes play a greater role, but anatomical circuits remain critical to understanding symptoms and developing therapies. In Parkinson disease, for example, drug and stimulation-based therapeutic interventions do not occur at the cellular lesion site, but rather are contingent on understanding interactions within the extra-pyramidal motor system [7]. Incomplete knowledge of this circuitry potentially holds back development of therapies for both Parkinson and Huntington diseases, despite a reasonably complete understanding of the genetic etiology of the latter.

There is growing evidence that aberrant wiring plays a central role in the etiology, pathophysiology, and symptomatology of schizophrenia [8], autism [9], and dyslexia [10]. Patients with autism and other pervasive developmental disorders are observed to have reductions in the size of the corpus callosum [11],[12] and in long-range frontal/temporal functional connectivity [13]–[15]. Autism is thought to be highly heritable and polygenic [16], and a number of mouse genetic models have been developed. The ability to compare the connectivity phenotypes of different mouse models with wild-type mice could yield important clues regarding the common pathways for generating the behavioral phenotype. Currently, however, the baseline connectivity data required to make such data-driven comparisons is lacking. If connectivity phenotypes can be established for autism and other disorders, these can assist in screening for drug development and more accurate subtyping of psychiatric diagnoses.

The importance of circuit considerations for differentially characterizing disorders such as major depression, anxiety, and obsessive–compulsive disorders, and substance (including nicotine) addiction is beginning to be recognized. These illnesses are considered disorders of the affective circuitry underlying emotion and motivated behaviors, which spans the brainstem, hypothalamus, frontal and cingulate cortices, and basal cortical nuclei [17],[18]. Knowledge of affective circuits is substantially poorer than of sensory–motor circuitry, despite disorders of the former resulting in a much greater burden of illness. Determining connectivity in these systems will allow the development of objective diagnostic tools, and may also yield cross-mammalian emotional endophenotypes to guide new conceptualizations of core psychiatric syndromes and aid drug discovery [19]. The development of animal models that mimic neuropsychiatric disorders at the circuit rather than behavioral level may also facilitate new therapeutic strategies. Furthermore, neuropsychiatric disorders likely result from pathologies at the system level, with complex genetic, epigenetic, and environmental factors combining to impact the neural circuitry. Systems-level knowledge, including neuroanatomic connectivity, may thus prove crucial in better understanding results from, for example, genomewide association studies. Analogously, the importance of incorporating knowledge from cellular systems biology (e.g., by grouping genes into pathways) has been recognized in other domains.

## What Is Being Proposed?

We propose a concerted experimental effort to comprehensively determine brainwide mesoscale neuronal connectivity in model organisms. Our proposal is to employ existing neuroanatomical methods, including tracer injections and viral gene transfer, which have been sufficiently well-established and are appropriately scalable for deployment at this level. The first and primary objective is to apply these methods in a standardized, high-throughput experimental program to fully map the mesoscale wiring diagram for the mouse brain and, following the model of successful genome projects, to rapidly make the results and digitized primary data publicly accessible. The second objective is to collate and, where possible, digitize existing experimental data from the macaque, and to pursue targeted experiments using standardized protocols to plug key gaps in our knowledge of primate brain connectivity. Additionally, we argue for similar efforts in other model organisms and for the pursuit of experimental methods that can be used in postmortem human brain tissue.

The projects may be carried out in a distributed manner by coordinating efforts at multiple experimental laboratories making use of uniform experimental protocols, or in a more centralized way by creating one or a few dedicated sites. Here we outline the properties of a large-scale connectivity mapping project that are seen as essential, and some that are desirable but not required. The required attributes are as follows.

1. *Brainwide coverage at a mesoscopic resolution*. The experimental technique must be applicable in all brain systems, cortical and subcortical. It should not be applicable only to specific cell types; if the technique is used to target specific cells, it must be capable of targeting any cell group.
2. *Validated and extensible experimental techniques*. The experimental methods must be well-characterized and, to the extent possible, validated. The *false positive* rate should be especially low. The techniques must be amenable to high-throughput application; the individual steps for sample preparation, injection, histology, detection, and data analysis should be stereotyped and of limited complexity.
3. *Centralized, open-access data repository*. The data collected from such an effort must be made freely available to all researchers from a centralized data repository. This includes raw image data, processed summary data, and metadata.
4. *Compatibility with existing neuroanatomical resources*. The results of this project must be interpretable with respect to existing datasets. For example, creating ties to the ABA [3], existing connectivity databases (Table 1), and other atlas projects (e.g., [20]–[22]) is imperative.
5. *Tractability with current informatics technology*. The data collected and maintained in the repository must be suitable to be analyzed and stored using existing informatics techniques and available technology, allowing for predictable growth in both methods and hardware.

**Table 1:** Databases and datasets containing information about neuroanatomical connections.

Database	Available Connectivity Information	URL
Brain Architecture Management System (BAMS) [2],[26]	Projections in rodent brain, curated manually from existing literature	http://brancusi.usc.edu/bkms/
Collations of Connectivity Data on the Macaque Brain (CoCoMac) [23],[24]	Projections in macaque brain, curated manually from existing literature	http://www.cocomac.org
Functional Anatomy of the Cerebro–Cerebellar System (FACCS) [29]	3D atlas of axonal tracing data in rat cerebro–cerebellar system	http://ocelot.uio.no/nesys/
BrainMaps.org [59]	Tables of connections from literature and primary data for some tracer injections	http://brainmaps.org
BrainPathways.org	Multiscale visualization of connectivity data from collated literature reports	http://brainpathways.org
Human Brain Connectivity Database	Curated reports of connectivity studies in postmortem human brain tissue	http://brainarchitecture.org
Internet Brain Connectivity Database	Estimated connectional data between human cortical gyral areas	http://www.cma.mgh.harvard.edu/ibcd/
Surface Management System DataBase (SumsDB) [64]	Connection densities from macaque retrograde tracer injections mapped to surface-based atlas	http://sumsdb.wustl.edu/sums/
SynapseWeb	Reconstructed volumes and structures from serial section electron microscopy	http://synapses.clm.utexas.edu/
Neocortical Microcircuit Database [71]	Connection data between single cells in mammalian cortex	http://microcircuit.epfl.ch/
ICBM DTI-81 Atlas [72]	Probabilistic atlas of human white matter tracts based on diffusion tensor imaging	http://www.loni.ucla.edu/Atlases/Atlas_Detail.jsp?atlas_id=15
Anatomy Toolbox Fiber Tracts [32]	Probabilistic atlas of human white matter tracts based on postmortem studies	http://www.fz-juelich.de/ime/spm_anatomy_toolbox
WormAtlas [30]	Full neuronal wiring data for *C. elegans*	http://www.wormatlas.org

Additional characteristics that would enhance the project's impact include the following.

1. *Availability of detailed anatomical information*. The ability to characterize various additional properties of the observed projection patterns would be beneficial. This might include classification of the neuronal cell types and neurotransmitters involved, laminar origins and terminations of projections in stratified structures, receptor information, cell density estimates in the origin and termination areas, morphological properties of the axons and/or dendritic arbors, and statistical characterization of topography and convergence or divergence patterns of projections.
2. *Reconstruction of projection trajectories*. In addition to the origins and terminations of projections, it would be valuable to determine their spatial trajectories. Such data would be particularly useful, for example, in understanding the impact of white matter lesions.
3. *Compatibility with high-resolution methods for targeted investigations*. While the primary imaging data should be obtained with light microscopy, electron microscopy or other high-resolution imaging methods could enable more detailed study of particular systems, provided the experimental protocols remain compatible with such techniques.
4. *Characterization of intersubject variability*. As discussed above, quantifying the variability of observed connectivity patterns would be valuable. This would require additional informatics methods and a substantially larger number of experiments than needed for estimating a single “map.”

## Where Are We Now?

Assessing the extent of current connectivity knowledge in various species is difficult because virtually all aspects of previous reports, including the specifics of animals used, experimental methodology, anatomical nomenclature, and presentation of results have varied across studies and laboratories. Furthermore, published data often include only processed results in the form of prose, tables, and schematic illustrations, while primary materials including original tissue sections sit on laboratory shelves.

A small number of public repositories for connectivity information are available (see Table 1), including two major efforts to manually curate reports for specific species. The CoCoMac (Collations of Connectivity data on the Macaque Brain) database catalogs axonal tracing studies from the monkey literature [23],[24] (approximately 400 literature reports detailing ∼2,800 tracer injections), while the Brain Architecture Management System (BAMS) focuses on the rat [25],[26] (328 references describing about 43,000 reported connections). Both systems organize connections based on discrete brain regions identified by the original authors according to a particular map or anatomical parcellation and use inference engines [2],[27],[28] to attempt to reconcile results across different parcellation schemes and nomenclature systems. These reconciliation processes possess considerable uncertainties, and the data remain very sparse; thus, any comprehensive picture of brain connectivity is not currently possible from such resources. The FACCS (Functional Anatomy of the Cerebro–Cerebellar System) database [29] is a strong effort to map connectivity *data* into a common spatial framework, but is currently limited in scope to the rat cerebrocerebellar system. Our understanding of the overall architecture of model nervous systems is currently limited to very simple organisms such as the nematode *Caenorhabditis elegans* [30].

Much of our theoretical knowledge of *human* brain connectivity comes from either very old sources [31] or from inference from varied reports in other species. Bürgel et al. [32] have developed a probabilistic atlas localizing major fiber bundles based on myelin staining in postmortem human brains, but these maps are very coarse and lack specificity in terms of termination zones. New technological developments such as diffusion-weighted magnetic resonance imaging (MRI) and computational techniques based on correlations in measured time series provide non-invasive methods for inferring some aspects of brain connectivity, but these methods necessarily require validation and should be complemented with more direct measurements. While an experimental program for the precise mapping of connectivity patterns in the *human* nervous system will require additional technological development, we are well-positioned to push forward with a systematic high-throughput experimental program for model organisms using mostly existing methods.

## A Survey of Available Techniques

Reviews of the history [33] and application of various techniques for determining anatomical connectivity can be found elsewhere [34],[35], and a further survey is presented in Text S1. Here we elaborate on methodologies suitable for the proposed experimental program.

*Neuronal tracers* allow injected molecules to be distributed within intact living neurons through active intra-axonal transport mechanisms. Tracer substances (see Text S1 for further details and properties) can be described by their preferred direction of transport, although labeling is often not exclusively unidirectional. Importantly, the majority of neuronal tracers can only be transported *within a cell* and do not cross the synapse; their utility in revealing the connectivity between brain areas is in tracing projection neurons either from axon terminals to potentially distant cell bodies, or vice versa. Retrograde transport (from axon terminal to cell body) is used to label the cells projecting to a particular target region, while anterograde transport (from cell bodies to axon terminals) allows for labeling the projection terminal regions of a cell or group of cells.

Modern “conventional” tracers yield strong, high-resolution labeling of fine processes, and can often be used in combination with one another, with histochemical techniques, genetic markers, light or electron microscopy, and a variety of delivery mechanisms. While there are many tracers that may prove suitable in a large-scale connectivity mapping project, *phaseolus vulgaris*–leucoagglutinin (PHA-L) [36] and high molecular weight (10 kDa) biotinylated dextran amines (BDA) [37],[38], both of which have now been used extensively and are transported primarily in the anterograde direction over sufficiently long distances, are strong candidates for high-throughput use. Either tracer can also be used in conjunction with a second high-resolution tracer such as cholera toxin subunit B (CTB) [39], which is transported primarily in the retrograde direction, in a multiple labeling protocol [40],[41]. Such multi-tracer methods allow a single experiment to be used to probe the inputs and outputs for a particular injection site at a relatively low additional cost in the detection process.

Some tracer substances, and in particular *neurotropic viruses* such as rabies virus [42] and the alpha herpes viruses [43],[44], can be transported transneuronally to label either presynaptic or postsynaptic cells. Viruses enter first-order neurons, replicate, and are transferred at or near the synapse to second-order cells where replication occurs again, thus continuing a self-amplification process. Viral spread, however, has a variable time course (which depends on projection strength), thus often making, for example, differentiation of weak first-order and strong second-order projections difficult, although this problem may be alleviated by using genetically engineered viruses that cross only a single synapse [45].

Replication-incompetent *viral vectors* engineered from adeno-associated virus (AAV), lentivirus, herpesvirus, and others can be used to drive high expression of fluorescent proteins as anterograde and retrograde tracers. These methods can have higher sensitivity than conventional tracer methods [46]–[48]. In addition, the number and types of infected neurons can be characterized, facilitating the pooling of data across multiple experiments. These viral reagents can be used in combination with transgenic mouse lines to label specific cell types [49]–[51]. It is clear that these and other genetic techniques will continue to gain prominence in neuroanatomy [52].

## How Will We Get There?

### Mouse

The first and primary phase of our proposal is to systematically map mesoscale connectivity in the mouse brain using standardized methods to label neuronal projections in combination with optical microscopy. The mouse, as opposed to rat, is the preferred rodent model due to its increasing use in neuroscience [53], the ease of use of transgenic methods, and the availability of large-scale spatial gene expression data in the brain [3],[22]. Accordingly, results from the mouse will be readily reconcilable with existing data, and new anatomical methods should be quickly applicable, supplying diverse information to supplement the initial experiments. A sample workflow, timeline, and cost estimates for a comprehensive mouse connectivity project are included in Text S2. We estimate that the complete mouse project can be completed in five years at a total cost of less than US$20 million, using five replicated experimental pipelines, each consisting of uniform experimental equipment with technicians implementing standardized protocols. Increasing the number of pipelines would proportionately reduce the timeline.

The proposed protocol calls for systematic injections of conventional tracers and/or viral vectors in the young adult mouse, age-matched and weight-matched to an existing stereotaxic brain atlas. The ABA has established a standard by using male, 56-day-old C57BL/6J mice [3], and this group has developed a corresponding anatomical reference atlas that is a reasonable choice to be adopted for this project. It is vital that the mouse strains, ages, and atlases used are common across the project. Furthermore, all surgical procedures, injection methods, histological techniques, and experimental apparatus should be uniform to reduce variability in results. The use of motorized stereotaxic manipulators with encoded positions relative to standard landmarks, and the incorporation of automation where possible within the experimental protocols will greatly aid this task. Equipment is now available for automated or semi-automated scanning and digitization of labeled sections at submicron resolution using fluorescence or bright field microscopy (see Text S1) and will form a critical piece of the experimental pipeline. Digitized images will be transferred into a distributed data-processing pipeline for automated analysis of the experimental results and entry into a public database.

The project will necessitate further development of algorithms to reliably extract wiring information from digitized images, and to bring data from different sections and animals into register with one another. Photomicrographs from an individual animal must be registered in 3D while accounting for tissue distortions, a process that can be improved by acquiring low-resolution reference block face images prior to cutting each section [54]. Detection of labeled cell bodies or clusters of cells and 3D registration to a Nissl-based atlas are problems that have been previously addressed on a large scale, for example, in the ABA [55]. Detection of labeled axonal segments is somewhat more challenging, and typically relies on (sometimes software-assisted) manual tracing, but progress has been made toward providing automated, quantitative estimates of axon length and density [56],[57]. Importantly, the objective of the analysis stream need not be to reconstruct individual neurons, but rather to detect and quantify labeled areas outside of the injection site and represent those data in a common framework (see also [58]).

### Primate

A high-throughput investigation in primates, on the scale proposed for mouse, is not feasible. Primate experiments are tremendously more costly, and the monkey brain is considerably larger, more complex, and more variable than mouse. It is therefore of critical importance that: 1) results from previous connectivity studies in primates are carefully curated from the existing literature, leveraging ongoing efforts such as CoCoMac [23],[24]; 2) efforts are made to systematically digitize slides that remain available from previous studies following such efforts as BrainMaps.org [59]; and 3) targeted experiments using standardized protocols are put in place that yield maximal data to validate and “fill in” the mesoscopic connectivity matrix for the macaque. See Text S3 for further details for a proposed primate connectivity project.

### Informatics Considerations

The success of the proposed efforts will hinge on the ability to coordinate activities across laboratories while maintaining quality control, to automate the analysis of acquired data, to store both raw and processed data, and to make the integrated results reliably available to different user groups through intuitive interfaces. Management of the large-scale dataset will require significant computational equipment and informatics expertise, some of which is likely to be distributed across multiple sites. The scope of the proposed project demands a customized laboratory information management system (LIMS) to organize and track tasks and materials within and across sites. Much can be learned from the informatics procedures carried out at the Allen Institute for Brain Science [55] and from the significant data-sharing efforts in genomics and bioinformatics [60],[61].

A major challenge is to develop an appropriate structured database to store the results of injection experiments, digitized legacy data, and associated metadata. In the CoCoMac and BAMS databases, the underlying data model of anatomy is discrete; that is, each “connection” is associated with a pair of discrete brain sites. Through systematic injections, and by preserving and storing primary image data, it is possible for the underlying data to be represented in analog form. Spatial databases [62] as used in geographical information systems and, in some cases, neuroscience [63] provide many of the necessary tools once the underlying data model (e.g., coordinate system) has been established. Anatomical parcellations based on different atlases may then be probabilistically registered to this coordinate space to enable the representation of the full connectivity data in the form of connectivity graphs or matrices, with “nodes” defined by the particular parcellation. The SumsDB database (http://sumsdb.wustl.edu/sums), for example, includes a surface-based macaque atlas containing many anatomical partitioning schemes registered to a common spatial framework, along with maps of neuronal connectivity from retrograde tracer injections registered from individual subjects to the atlas [64]. Representation in the continuous space additionally allows for a post-hoc analysis that *solves for* the partitioning of brain space that best follows the connectivity patterns observed in the data.

### Technology Development and Evaluation for Human Studies

The ultimate goal of our proposal to experimentally map brainwide connectivity patterns is to arrive at a comprehensive understanding of the architecture of the *human* brain. A much-improved partial understanding can be obtained from the proposed efforts in mouse and macaque, and a proposal has been made for a human connectivity project that would rely primarily on neuroimaging techniques [65]. Still, resources should be devoted to developing classical neuroanatomical techniques that are viable for humans. There have been sporadic efforts to increase the speed of action for lipophilic carbocyanine dyes when used in fixed postmortem human tissue [66],[67], and these and other efforts should be studied further. Additionally, imaging methods including diffusion tensor imaging and diffusion spectrum imaging, as well as computational techniques for the assessment of “functional” or “effective” connectivity [68] can be validated by supplementing tracer studies in macaque with MRI data acquisition in the same animals. Such efforts are essential to ultimately reversing the backwardness of human neuroanatomy.

## Conclusions

The largest current gap that exists between the genotype and phenotype in neuroscience is at the level of brain connectivity. There is thus enormous potential value in the acquisition of comprehensive, unified connectivity maps in model organisms. We have proposed a concerted effort within the neuroscience community to determine these connectivity patterns *brainwide* at the tractable yet representative mesoscopic scale, first in the mouse and followed by additional efforts for the macaque and eventually humans. The mouse proposal is based on existing methods, scaled up, and standardized for high-throughput experimentation. This effort would be complementary to, and would provide “scaffolding” for, additional anatomical projects using different emerging technologies, and can be integrated with existing resources such as the ABA to probe various levels of structural and functional organization. Examination of a potential project plan demonstrates that such an effort would be relatively inexpensive in terms of both money and time (see Text S2) compared with its potential value in neuroscience and biomedicine. If successful, similar projects could be undertaken for model organisms including vertebrates such as rat, zebrafish, zebra finch, and chick, and invertebrates such as jellyfish, flatworms, and drosophila, enabling comparative neuroanatomical studies that are currently well beyond reach.

While the principal objective of the proposed project is to characterize and make available a “wiring diagram,” the public availability of raw data is vital to allow researchers to form their own, perhaps more detailed, interpretation of the individual results. Technological advances have only recently made it feasible to capture and store the voluminous raw image data at submicron resolution, and to serve these images over the Web. The spirit of collaboration and open data access requisite in this proposal is also currently reflected in increasing proportions within the neuroscience community and within funding agencies, as reflected, for example, in the NIH Blueprint for Neuroscience Research [69] and in international neuroinformatics initiatives [70]. Thus, we may be at a point in time that makes a project of this sort uniquely feasible. Realizing the vision put forth here will require additional planning, input from the community, and financial support. Moreover, eventually determining the connectivity matrix for *human* will require additional technical development. The hope, however, is that this proposal has made both the importance and the viability of brainwide connectivity projects apparent, and that we can move from planning to action on a short timescale.

## Supporting Information

Text S1 Survey of methods relevant for determining neuronal connectivity. To supplement the discussion provided in the main article, here we provide a brief general overview of experimental methods for determining and imaging neuronal connection patterns.
(0.34 MB PDF)

Text S2 Example workflow, informatics requirements, timeline, and cost estimates for mouse connectivity project. Here we describe in greater detail a possible experimental pipeline and data analysis workflow for a systematic study of mesoscale mouse brain connectivity using neuroanatomical tracers.
(1.43 MB PDF)

Text S3 Brief proposal for primate connectivity project. We describe an approach to better understand connectivity in the macaque brain that includes collating and digitizing existing materials as well as implementing specifically targeted experiments with standard protocols.
(0.15 MB PDF)
