# Supplementary material for: A Proposal for a Coordinated Effort for the Determination of Brainwide Neuroanatomical Connectivity in Model Organisms at a Mesoscopic Scale
Source: PLoS Comput Biol. 2009 Mar 27;5(3):e1000334. doi: 10.1371/journal.pcbi.1000334 (PMC2655718; doi:10.1371/journal.pcbi.1000334)
Supplement: Text S1 — Survey of methods relevant for determining neuronal connectivity. To supplement the discussion provided in the main article, here we provide a brief general overview of experimental methods for determining and imaging neuronal connection patterns. (0.34 MB PDF) [file pcbi.1000334.s001.pdf]

A proposal for a coordinated effort for the determination of brainwide neuroanatomical connectivity in model organisms at a mesoscopic scale: Supporting information

Text S1: Survey of methods relevant for determining neuronal connectivity

To supplement the discussion provided in the main article, here we provide a brief general overview of experimental methods for determining and imaging neuronal connection patterns.

Classical methods: The most basic class of anatomical methods aimed at connectivity, which are based on gross dissection, have been used roughly since the initial distinction between white and grey matter was made by Vesalius [1], with the "teasing method" for following the course of fiber bundles developed by Steno in the 17th century effectively inaugurating the era of tract tracing. Gross dissection provided a vehicle for an elementary understanding of white matter structures, but is severely limited by an overall lack of precision in defining anything but the largest pathways and an inability to map the fibers to their precise terminations.

Innovations in staining methods [2] as well as the development of neuronal degeneration techniques [3,4] led to Dejerine's comprehensive study of the human white matter pathways, still frequently referenced today [5]. In later years such methods were improved to selectively stain degenerating fibers [6,7], and these techniques have provided much of what is known even today about the fiber systems in humans. Still, degeneration methods are severely limited due to a lack of selectivity, and because they require insult to the neural tissue under investigation. Furthermore these methods are tedious and labor-intensive and are not feasible for any large-scale attempt to determine brainwide connectivity patterns at an appropriate level of resolution.

Diffusion and functional imaging: Non-invasive brain imaging techniques are now the most common tools for modern *human* neuroanatomy. Diffusion tensor imaging (DTI) [8] can be used to estimate the tensor describing water diffusion at each voxel in a magnetic resonance image (MRI) volume. This technique is of use for assessing connectivity because diffusion within the white matter is constrained by axonal membranes with particular orientations. Various tractography algorithms can be used to "connect" the tensors along their principal directions to give an estimate of the orientations of fibers traversing through the volume. Studies have demonstrated that such techniques can yield the approximate trajectories of a number of major fiber bundles that have been demonstrated via other methods in either human or non-human primates [9,10]. DTI, however, is limited in its specificity where fibers enter the gray matter, and also because of considerable ambiguity at potential crossings of fiber tracts within the volume. Diffusion spectrum imaging (DSI) [11] reduces the problem of fiber crossings by providing a much finer sampling of the orientation distribution function at each voxel.

Another class of inferential methods is based on analysis of correlations in multivariate time series data obtained from either direct measurements using, for example, multi-unit electrodes, or from indirect measurements using electro- or magnetoencephalography (EEG/MEG) or functional MRI. A wide variety of statistical techniques are now available to infer functional networks from these correlations [12,13,14,15], yet the inference of *anatomical* connectivity from task-based functional data is tenuous. Indeed the availability of mesoscopic connectivity information would greatly constrain efforts to measure task-related modulations of functional or effective connectivity in imaging studies. There has been recent interest in spatially correlated activity observed in *resting state fMRI* [16,17,18] where the subject is not actively performing a task. Results suggest that spontaneous correlated spatial patterns may reflect anatomical

subdivisions [19], but further data are needed to understand the complex relationship between correlations in the blood oxygenation level dependent (BOLD) response and physical connections between the neurons indirectly giving rise to that response. Still, brain imaging methods provide truly non-invasive techniques for the study of connectivity, especially in humans, and may prove quite valuable, particularly for longitudinal studies. An important contribution of a large-scale connectivity project using other more well-established methods in non-humans can be to help validate these methods (see the primate project described in Text S3).

Neuronal tracers: A great advancement in neuroanatomy occurred as tracers based on axonal transport were rapidly introduced beginning in the 1970s, allowing injected molecules to be distributed within intact living neurons through active intra-axonal transport mechanisms. In their typical use, following a variable post-injection survival period, the animal is sacrificed, the brain tissue processed, and tracer detected through histochemical reactions and microscopy. The versatility of neuronal tracers has made them the primary method for examining anatomical connectivity over the past several decades. The first tracers used radiolabeled amino acids [20] incorporated into cell proteins and transported in the anterograde direction. The transported radiolabeled isotopes can then be detected using autoradiography. This method has been used extensively in monkey studies, but suffers from an indirect labeling method and a lack of resolution compared with more recent tracers. Retrograde transport was achieved by injection of the enzyme horseradish peroxidase (HRP) [21,22], which could be robustly detected with a simple histochemical reaction using, for example, diaminobenzidine (DAB) or tetramethylbenzidine (TMB) as a substrate.

More recently many improved "conventional" tracer substances that result in stronger or higher-resolution labeling have been developed (see Table 1). We discuss two such tracer substances with preference for anterograde transport in the main article and in more detail presently. *Phaseolus vulgaris* – leucoagglutinin (PHA-L) [23] is a lectin derived from red kidney beans that binds to glycoproteins in neuronal membranes and is taken up into cell bodies and is transported almost exclusively in the anterograde direction. Biotinylated dextran amines (BDA) [24,25] are dextrans, which have been used in other forms as tracers, that are conjugated to biotin. Available in different molecular weights, the heavier versions (e.g. BDA10k; 10 kDa MW) appear to show stronger preference for anterograde transport, whereas retrograde transport is observed for lighter versions [26]. Still BDA is not transported in either direction exclusively, which can lead to some ambiguity in interpretation of labeling results; this problem is minimized with PHA-L. The incorporation of biotin in BDA enables a simple histochemical detection procedure that is suitable for light or electron microscopy using a standard avidin-biotinylated HRP (ABC) kit followed by DAB reaction, whereas the detection of PHA-L requires incubation in primary and secondary antibodies. Both can be injected by iontophoresis, preferred for small injection sites, or by pressure injection for larger targets.

We have suggested that the use of a multi-tracer protocol [27,28] involving co-injection of an anterograde and retrograde tracer may prove beneficial in a high-throughput experimental program. The non-toxic B subunit of cholera bacterial toxin (CTB) [29] is a strong candidate for use as such a (primarily) retrograde tracer. Alternatively, the fluorescent tracer Fluoro-gold [30] is also suitable for combination with other tracers in rodents [31]. Multiple labeling protocols

require that each tracer be revealed as a distinct reaction product; this can be easily achieved with an appropriately designed series of standardized histochemical processes.

It is outside of our purposes to discuss the merits of each neuronal tracer (though see Table 1), but another class – the lipophilic carbocyanine dyes (including DiI, DiO, and DiA) – provide at least one property not available to the majority of other methods: the ability to act in fixed post-mortem human brain tissue through passive diffusion [e.g. 32]. While the slow diffusion process has thus far limited their use to the study of relatively short-range (up to 2-3 cm) projections, attempts have been made to increase the speed of transport to ultimately make the method more viable for human tissue [33,34]. These and other fluorescent tracers can be injected *in vivo* as well, but have few benefits relative to the other tracers discussed above, and suffer from fading of the fluorescence signal after exposure to light. The labeling can be made into a permanent substrate through photoconversion, but the process is time consuming and not viable for high-throughput application.

Trans-neuronal tracers: Some tracer substances can be transported trans-neuronally by crossing the synaptic cleft to label either pre- or post-synaptic cells. Among "conventional" tracers these include the non-toxic C fragment of tetanus (TTC), wheat germ agglutinin (WGA) and, in some systems, WGA conjugated to horseradish peroxidase (WGA-HRP). These tracers become increasingly dilute as they spread, resulting in the problem of weak labeling and difficult detection. The use of neurotropic viruses has largely superseded the utilization of such tracers for labeling multi-synaptic pathways (see Table 2). The two major classes of viruses in use as trans-neuronal tracers are rabies virus [35] and the alpha herpes viruses [36,37] including the swine pseudorabies virus (PRV; not related to rabies virus) and herpes simplex virus (HSV-1). Viruses enter the cell bodies of first-order neurons, replicate, and are then transferred at or near the synapse to second-order cells where replication occurs again. The virus thus has the important property of self-amplification, which results in generally superior labeling of neurons compared to the non-viral trans-neuronal tracers. Because virus spread across multiple synapses has a variable time course (which is affected by the strength of projections, for example), it is often difficult to differentiate weak first order connections from strong second-order projections, and so on. For this reason it is typically necessary to evaluate viral labeling (using immunohistochemical procedures) at multiple time points following injection, requiring additional injections and additional animals.

Transgenesis and viral gene delivery: One of the primary criticisms of conventional tracer techniques is their general inability to target specific cell types. The incorporation of genetic methods into the neuroanatomist's toolbox [reviewed in Refs. 38,39] has begun to bridge this gap as well as to augment the conventional techniques. Here we give only a few illustrations of these techniques. For example, WGA cDNA has been employed as a transgene in mice, with expression under the control of cell-type specific promoters [40,41] in order to label selective multi-synaptic circuits in the anterograde direction. TTC fused with green fluorescent protein (GFP) expression has similarly been engineered in transgenic mice for retrograde tracing [42]. These genetically targeted trans-neuronal tracers still, however, suffer from the same shortcomings of their conventional counterparts, particularly low sensitivity to weak connections.

Viral tracers have additionally benefited from genetic manipulation. The PRV virus was engineered to be cell-type specific by replacing the coding sequence for thymidine kinase (TK), a gene necessary for replication, with a conditionally expressed TK gene as well as the coding sequence for GFP [43]. The modified virus then is only able to replicate in cells expressing Cre recombinase, and these cells also express the fluorescent protein. When the virus is recombined, it spreads as usual in the retrograde direction, with infected cells in the multi-synaptic pathway also expressing GFP. Thus this method allows the initial first-order target of the virus to be restricted to cell populations that themselves can be targeted for Cre expression through transgenic methods. A recent innovation was made by Wickersham et al. [44] to restrict trans-synaptic labeling by a deletion mutant rabies virus to a single synapse, beginning from a targeted cell population. The virus envelope protein (rabies glycoprotein; RG), necessary for trans-synaptic spread but not for transcription or virus replication, was replaced by enhanced green fluorescent protein (EGFP). The missing virus glycoprotein gene is then supplied *in trans* to a cell group targeted for initial infection with the mutant tracer virus. The result is that the virus can assemble and spread trans-synaptically from this initial cell population, but because RG is not present in the pre-synaptic cells, the virus can not continue to spread. Infected cells also strongly express EGFP making detection with fluorescence microscopy straight-forward.

Replication incompetent viral vectors engineered from adeno-associated viruses (AAV), lentivirus, and others can be used to deliver genetic material to selected cells [45], notably to drive high expression of fluorescent proteins as anterograde and retrograde tracers. Recombinant AAV [46] and lentivirus [47,48] have both, for example, been used to deliver GFP to cells by injection, resulting in a robust anterograde labeling of axons over potentially long distances that is stable over time. This method thus acts much like a "conventional" anterograde tracer, with potentially higher sensitivity, and can be visualized with fluorescence microscopy or with bright-field microscopy after appropriate antibodies. Additionally lentivirus has been engineered with GFP-tagged synaptophysin to produce selective labeling at presynaptic terminals [47]. These viral vectors can additionally be used in combination with transgenic mouse lines to target specific cell types [49,50].

Recently *Brainbow* transgenic mice were developed also exploiting the Cre-Lox system [51]. Through engineered combinatorial expression of multiple fluorescent proteins, targeted neurons take on many (up to 90 in the published report) distinct color profiles. The color variations allow one to distinguish adjacent neurons and thus to limit ambiguity in the reconstruction of axons and synaptic contacts. While not a tracer method, the *Brainbow* construct offers the ability to probe detailed microcircuitry in relatively large groups of labeled neurons; furthermore automation of neuron reconstruction should be made easier by the distinct color profiles, which have been shown to be relatively stable within each cell.

Microscopy methods: Any of the neuronal tracing techniques available require a stage in which labeled cell bodies or neuronal processes are revealed through an imaging process. This has been typically achieved through light microscopy (LM), which current remains the most viable option for a high-throughput setting. Image capture for the Allen Institute's gene expression atlas project was fully automated using Leica DM6000B microscope systems (http://www.leica-microsystems.com/) [52], and the Brainmaps.org project [53] has used systems from Aperio Technologies (http://www.aperio.com) for "virtual microscopy." These types of automated

imaging technologies, which have only become available in recent years, can offer very high (less than 1 square micron) in-plane image resolution and will be suitable for detection of reaction products resulting from immunohistochemistry. In addition to systems from Leica and Aperio, the NanoZoomer from Olympus (http://www.olympusamerica.com/), and the Mirax Scan System from Carl Zeiss, Inc. (http://www.zeiss.com) offer similar technologies.

Such LM technologies, however, are insufficient for extremely high-resolution imaging of synapses and receptors or other subcellular organelles. Recent developments in ultrastructural imaging including array tomography, serial reconstruction of pathways[54] at the site of termination [e.g. Ref. 55], serial block-face scanning electron microscopy (SBFSEM), and the automatic tape-collecting lathe ultra-microtome (ATLUM), have opened the possibility of obtaining accurate large volume images at nanometer resolution [56,57,58]. Such methods are not tractable for comprehensive brainwide mapping in large brains, and it is unclear that the required neuron reconstruction algorithms ready for high-throughput usage. Further, some elements of microcircuit organization may be inferred without ultrastructural reconstructions using principles such as Peter's Rule [59]. However, these techniques should be used to provide detailed supplemental data to the proposed project. For this reason it is important that techniques applied to a brainwide project remain compatible with EM.

coarser → finer

Procedure	Used in	Spatial specificity	Brief Description
Gross dissection (1543)	postmortem human	major fiber bundles	classical “hands on” anatomy
Myelin stains (1882)	any species	large and fine myelinated axon bundles	ferric chloride and hematoxylin stain myelinated fibers deep blue
Degeneration Methods (1885-1950’s)	any species	degenerating axon bundles	e.g. silver impregnation selectively labels degenerating fibers from a lesion site
Diffusion-based MR imaging (1990 -)	primates including human	major fiber bundles	MR imaging of water diffusion direction + reconstruction
Neuronal tracers (1968 -)	rodents, primates, postmortem human	Single neurons and/or fibers down to neural processes	active or passive transport of injected substances
Transgenesis (1990 -)	mice, drosophila	fiber bundles down to neural processes	selective expression of genes to label specific cells
Viral gene transfer (1998 -)	rodents, non-human primates	fiber bundles down to neural processes	selective expression of genes to labels specific cells
SPIM / Ultramicroscopy (2007)†	rodents	single cell	planar imaging of whole sample
Volume EM reconstruction (2004 -)†	any species	nanometer resolution; single boutons, spines, receptors and other subcellular organelles	serial scanning of ultrathin sections, or block-face scanning of three-dimensional volumes

Table 1: Techniques for determining neuroanatomical connectivity. † Imaging methods (not connectivity methods *per se*) that can be used in conjunction with dyes and tracers, etc. for detailed reconstruction of microcircuitry.

	Labeling efficacy										
Tracer	A	R	Resolution	Application	Detection	Visual-ization	Mechanism	Trans-synaptic	Transport rate	Preparations	Limitations
Radiolabeled amino acids[20]			Medium	S	Autoradiography	LM, DFM	Protein synthesis and fast axonal transport		up to 100 mm/day	In vivo primate, rodent	Inferential detection method
Horseradish peroxidase (HRP)[21,22,60]			Medium-Fine	P, I, S	ABC method	LM, EM	Endocytotic uptake, retrograde vesicular transport		200-300 mm/day	In vivo rodent, primate	Tracer leakage, lack of fine labeling
Lipophilic Dyes (DiI, FastDiI, DiA, DiO)[61]			Medium-Fine	P, I, S, C, B	Red, green, blue fluorescence, photoconversion	FM, LM	Lateral diffusion within fluid membrane		up to 6 mm/day in vivo, ~2 mm/mo in fixed tissue	In vivo rodent, primate, slice cultures, fixed tissue, birds	Photobleaching, failure in adult animals
Fluoro-Gold (FG)[30]			Fine	P, I, C	gold-blue fluorescence, photoconversion, IH	FM, LM, EM	Endocytotic uptake, retrograde vesicular transport			In vivo rodent	Limited use for long-term studies; cytotoxicity
Phaseolus vulgaris-leucoagglutinin (PHA-L)[23]			Fine	I	IH	LM, EM	Binds to cell surface receptors		4-7 mm/day	In vivo rodent	Not effective in older animals, unreliable for some researchers in primates
Wheat germ agglutinin (WGA)[62]			Medium	P, I, T	IH	LM, EM	Binds to cell surface receptors	Y	22-44 mm/day	In vivo rodent, primate, transgenic mice	Severe immune response, weak transneuronal labeling
Cholera toxin subunit B (CTB)[63]			Fine	P, I	IH	LM, EM	Binds to cell surface receptors		~100 mm/day	In vivo rodent, primate, birds	Some anterograde transport
C fragment of tetanus toxin (TTC)[64]			Medium	P,I	IH	LM, EM		Y	~75 mm/day	In vivo rodent, primate	weak transneuronal labeling
Biotinylated dextran amines[24,25] (BDA 10kDa)			Fine	P, I	ABC method	LM, EM	Probably endocytotic uptake followed by diffusion		2-6 mm/day	In vivo rodent, primate	Some retrograde transport through fibers of passage
Biotinylated dextran amines[24,25] (BDA 3kDa)			Fine	P, I	ABC method	LM, EM	Probably endocytotic uptake followed by diffusion		2-6 mm/day	In vivo rodent, primate	Some anterograde transport
Fluorescent dextrans (FR, FE, CB, LY)[65,66]			Fine	P, S	Multi-color Fluorescence, photoconversion, IH	FM, LM, EM	Probably endocytotic uptake followed by diffusion			In vivo rodent, primate	Photobleaching of label
Biocytin / Neurobiotin[67]			Fine	IC, P, I	ABC method	LM, EM	Fast axonal transport		36-72 mm/day	In vivo rodent	Only useful for short survival periods
Fluorescent latex microspheres[68]			Medium	P, S	Multi-color Fluorescence	FM, LM	Retrograde vesicular transport			In vivo rodent, slice culture	No cell morphology visible, limited compatibility with histological methods

Table 2: Comparison of conventional neuronal tracer substances. In columns titled *Labeling efficacy*, subcolumn A indicates anterograde direction, R retrograde direction, and the gray-level indicates efficacy with black strongest. In column *Application,* S: Hamilton microsyringe, P: pressure injection, I: iontophoresis, C: crystal placement, B: biolistic delivery, T: transgenic mice. Other acronyms: IH: immunohistochemistry, LM: light microscopy, FM: fluorescence microscopy, EM: electron microscopy, DFM: dark field microscopy, ABC method: avidin biotinylated HRP method.

Virus	Direction		Specific targets	Trans-synaptic	Reporter Method	Genetic constructs required	Human pathogen	Known issues / considerations
	A	R						
alpha-herpesviruses								
swine pseudorabiesvirus (PRV)								PRV viruses fail to work in primates, virus may not exclusively spread to synaptically connected cells
PRV-Becker				multiple	IH	N	N	highly virulent wild type strain, not viable as tracer due to short survival times
PRV-Kaplan				multiple	IH	N	N	
PRV-Bartha				multiple	IH	N	N	immune reactivity, polysynaptic, distribution of receptors, ventricle uptake
PRV152[69]				multiple	EGFP	N	N	
PRV614[70]				multiple	RFP	N	N	
PRV-BaBlue[71]				multiple	β-Gal	N	N	
Ba2001[43]			Cre expressing cells	multiple	EGFP	Y	N	
human herpes simplex virus (HSV)								Herpes viruses can infect astrocytes as well as neurons
HSV-1 (MacIntyre)				multiple	IH	N	Y	Transport direction may differ by infected area
HSV-1 (H129)[72]				multiple	IH	N	Y	
HSV-2 (Strain 186)				multiple	IH	N	Y	Transport direction may differ by infected area
rhabdoviruses								
Challenge Virus Standard (CVS-11)[35]				multiple	IH	N	Y	May not work in all systems in all species
Deletion-mutant rabies virus[44]			TVA expressing cells	single	GFP, RFP	Y	Y	Not currently available *in vivo*
Recombinant Adeno-associated virus (rAAV)[46]				none	IF	N	N	
Recombinant Lentivirus[47]				none	GFP, IF	N	N	

Table 3: Summary of a subset of the viral tracers (including recombinant viruses) that have been utilized.

References

1. Vesalius A (1543) De humani corporis fabrica libri septem. Basel: J. Oporinus.
2. Weigert C (1884) Ausfuhrliche beschreibung der in No. 2 dieser Zeitschrift erwahnten neuen Farbungsmethode fur das centralnervensystem. Fortschr Med 2: 190-191.
3. Marchi V, Algeri G (1885) Sulle degenerazioni discendenti consecutive a lesioni della corteccia cerebrale. Revista sperimentale di freniatria e medicina legale in relazione con l'antropologia e le scienze giuridiche e sociali Reggio-Emilia 11: 492Â–494.
4. Waller A (1850) Experiments on the Section of the Glossopharyngeal and Hypoglossal Nerves of the Frog, and Observations of the Alterations Produced Thereby in the Structure of Their Primitive Fibres. Philosophical Transactions of the Royal Society of London 140: 423-429.
5. Dejerine J (1895) Anatomie des centres nerveux. Paris: Rueff.
6. Fink RP, Heimer L (1967) Two methods for selective silver impregnation of degenerating axons and their synaptic endings in the central nervous system. Brain Res 4: 369-374.
7. Nauta WJ, Gygax PA (1954) Silver impregnation of degenerating axons in the central nervous system: a modified technic. Stain Technol 29: 91-93.
8. Le Bihan D, Mangin JF, Poupon C, Clark CA, Pappata S, et al. (2001) Diffusion tensor imaging: concepts and applications. J Magn Reson Imaging 13: 534-546.
9. Makris N, Kennedy DN, McInerney S, Sorensen AG, Wang R, et al. (2005) Segmentation of subcomponents within the superior longitudinal fascicle in humans: a quantitative, in vivo, DT-MRI study. Cereb Cortex 15: 854-869.
10. Mori S, Wakana S, Van Zijl PCM (2005) MRI Atlas of Human White Matter: Elsevier Science.
11. Wedeen VJ, Hagmann P, Tseng WY, Reese TG, Weisskoff RM (2005) Mapping complex tissue architecture with diffusion spectrum magnetic resonance imaging. Magn Reson Med 54: 1377-1386.
12. Bressler SL, Richter CG, Chen Y, Ding M (2007) Cortical functional network organization from autoregressive modeling of local field potential oscillations. Stat Med 26: 3875-3885.
13. Friston KJ, Harrison L, Penny W (2003) Dynamic causal modelling. Neuroimage 19: 1273-1302.
14. McIntosh AR, Grady CL, Ungerleider LG, Haxby JV, Rapoport SI, et al. (1994) Network analysis of cortical visual pathways mapped with PET. J Neurosci 14: 655-666.
15. Roebroeck A, Formisano E, Goebel R (2005) Mapping directed influence over the brain using Granger causality and fMRI. Neuroimage 25: 230-242.
16. Fox MD, Raichle ME (2007) Spontaneous fluctuations in brain activity observed with functional magnetic resonance imaging. Nat Rev Neurosci 8: 700-711.
17. Fox MD, Snyder AZ, Vincent JL, Corbetta M, Van Essen DC, et al. (2005) The human brain is intrinsically organized into dynamic, anticorrelated functional networks. Proc Natl Acad Sci U S A 102: 9673-9678.
18. Vincent JL, Patel GH, Fox MD, Snyder AZ, Baker JT, et al. (2007) Intrinsic functional architecture in the anaesthetized monkey brain. Nature 447: 83-86.
19. Cohen AL, Fair DA, Dosenbach NU, Miezin FM, Dierker D, et al. (2008) Defining functional areas in individual human brains using resting functional connectivity MRI. Neuroimage 41: 45-57.
20. Cowan WM, Gottlieb DI, Hendrickson AE, Price JL, Woolsey TA (1972) The autoradiographic demonstration of axonal connections in the central nervous system. Brain Res 37: 21-51.
21. Lavail JH, Lavail MM (1972) Retrograde Axonal Transport in the Central Nervous System. Science 176: 1416-1417.
22. Kristensson K, Olsson Y (1971) Retrograde axonal transport of protein. Brain Res 29: 363-365.
23. Gerfen CR, Sawchenko PE (1984) An anterograde neuroanatomical tracing method that shows the detailed morphology of neurons, their axons and terminals: immunohistochemical localization of an axonally transported plant lectin, Phaseolus vulgaris leucoagglutinin (PHA-L). Brain Res 290: 219-238.
24. Reiner A, Veenman CL, Medina L, Jiao Y, Del Mar N, et al. (2000) Pathway tracing using biotinylated dextran amines. J Neurosci Methods 103: 23-37.
25. Veenman CL, Reiner A, Honig MG (1992) Biotinylated dextran amine as an anterograde tracer for single- and double-labeling studies. J Neurosci Methods 41: 239-254.
26. Raju DV, Smith Y (2006) Anterograde axonal tract tracing. Curr Protoc Neurosci Chapter 1: Unit 1 14.

27. Coolen LM, Jansen HT, Goodman RL, Wood RI, Lehman MN (1999) A new method for simultaneous demonstration of anterograde and retrograde connections in the brain: co-injections of biotinylated dextran amine and the beta subunit of cholera toxin. J Neurosci Methods 91: 1-8.
28. Lanciego JL, Luquin MR, Guillen J, Gimenez-Amaya JM (1998) Multiple neuroanatomical tracing in primates. Brain Res Brain Res Protoc 2: 323-332.
29. Luppi PH, Fort P, Jouvet M (1990) Iontophoretic application of unconjugated cholera toxin B subunit (CTb) combined with immunohistochemistry of neurochemical substances: a method for transmitter identification of retrogradely labeled neurons. Brain Res 534: 209-224.
30. Schmued LC, Fallon JH (1986) Fluoro-Gold: a new fluorescent retrograde axonal tracer with numerous unique properties. Brain Res 377: 147-154.
31. Risold PY, Swanson LW (1995) Evidence for a hypothalamothalamocortical circuit mediating pheromonal influences on eye and head movements. Proc Natl Acad Sci U S A 92: 3898-3902.
32. Burkhalter A, Bernardo KL (1989) Organization of corticocortical connections in human visual cortex. Proc Natl Acad Sci U S A 86: 1071-1075.
33. Sparks DL, Lue LF, Martin TA, Rogers J (2000) Neural tract tracing using Di-I: a review and a new method to make fast Di-I faster in human brain. J Neurosci Methods 103: 3-10.
34. Swift MJ, Crago PE, Grill WM (2005) Applied electric fields accelerate the diffusion rate and increase the diffusion distance of DiI in fixed tissue. J Neurosci Methods 141: 155-163.
35. Kelly RM, Strick PL (2000) Rabies as a transneuronal tracer of circuits in the central nervous system. J Neurosci Methods 103: 63-71.
36. Aston-Jones G, Card JP (2000) Use of pseudorabies virus to delineate multisynaptic circuits in brain: opportunities and limitations. J Neurosci Methods 103: 51-61.
37. Ekstrand MI, Enquist LW, Pomeranz LE (2008) The alpha-herpesviruses: molecular pathfinders in nervous system circuits. Trends Mol Med 14: 134-140.
38. Callaway EM (2005) A molecular and genetic arsenal for systems neuroscience. Trends Neurosci 28: 196-201.
39. Luo L, Callaway EM, Svoboda K (2008) Genetic dissection of neural circuits. Neuron 57: 634-660.
40. Yoshihara Y (2002) Visualizing selective neural pathways with WGA transgene: combination of neuroanatomy with gene technology. Neurosci Res 44: 133-140.
41. Yoshihara Y, Mizuno T, Nakahira M, Kawasaki M, Watanabe Y, et al. (1999) A genetic approach to visualization of multisynaptic neural pathways using plant lectin transgene. Neuron 22: 33-41.
42. Maskos U, Kissa K, St Cloment C, Brulet P (2002) Retrograde trans-synaptic transfer of green fluorescent protein allows the genetic mapping of neuronal circuits in transgenic mice. Proc Natl Acad Sci U S A 99: 10120-10125.
43. DeFalco J, Tomishima M, Liu H, Zhao C, Cai X, et al. (2001) Virus-assisted mapping of neural inputs to a feeding center in the hypothalamus. Science 291: 2608-2613.
44. Wickersham IR, Lyon DC, Barnard RJ, Mori T, Finke S, et al. (2007) Monosynaptic restriction of transsynaptic tracing from single, genetically targeted neurons. Neuron 53: 639-647.
45. Davidson BL, Breakefield XO (2003) Viral vectors for gene delivery to the nervous system. Nat Rev Neurosci 4: 353-364.
46. Chamberlin NL, Du B, de Lacalle S, Saper CB (1998) Recombinant adeno-associated virus vector: use for transgene expression and anterograde tract tracing in the CNS. Brain Res 793: 169-175.
47. Grinevich V, Brecht M, Osten P (2005) Monosynaptic pathway from rat vibrissa motor cortex to facial motor neurons revealed by lentivirus-based axonal tracing. J Neurosci 25: 8250-8258.
48. Dittgen T, Nimmerjahn A, Komai S, Licznerski P, Waters J, et al. (2004) Lentivirus-based genetic manipulations of cortical neurons and their optical and electrophysiological monitoring in vivo. Proc Natl Acad Sci U S A 101: 18206-18211.
49. Kuhlman SJ, Huang ZJ (2008) High-resolution labeling and functional manipulation of specific neuron types in mouse brain by Cre-activated viral gene expression. PLoS ONE 3: e2005.
50. Atasoy D, Aponte Y, Su HH, Sternson SM (2008) A FLEX switch targets Channelrhodopsin-2 to multiple cell types for imaging and long-range circuit mapping. J Neurosci 28: 7025-7030.
51. Livet J, Weissman TA, Kang H, Draft RW, Lu J, et al. (2007) Transgenic strategies for combinatorial expression of fluorescent proteins in the nervous system. Nature 450: 56-62.
52. Ng L, Pathak SD, Kuan C, Lau C, Dong H, et al. (2007) Neuroinformatics for genome-wide 3D gene expression mapping in the mouse brain. IEEE-ACM Transactions on Computational Biology and Bioinformatics 4: 382-393.

53. Mikula S, Trotts I, Stone JM, Jones EG (2007) Internet-enabled high-resolution brain mapping and virtual microscopy. Neuroimage 35: 9-15.
54. Fiala JC (2005) Reconstruct: a free editor for serial section microscopy. J Microsc 218: 52-61.
55. Zikopoulos B, Barbas H (2007) Parallel driving and modulatory pathways link the prefrontal cortex and thalamus. PLoS ONE 2: e848.
56. Blow N (2007) Following the wires. Nat Meth 4: 975-981.
57. Briggman KL, Denk W (2006) Towards neural circuit reconstruction with volume electron microscopy techniques. Curr Opin Neurobiol 16: 562-570.
58. Micheva KD, Smith SJ (2007) Array tomography: a new tool for imaging the molecular architecture and ultrastructure of neural circuits. Neuron 55: 25-36.
59. Braitenberg V, Schüz A (1991) Anatomy of the cortex: statistics and geometry: Springer-Verlag, Berlin; New York.
60. Mesulam MM (1982) Tracing neural connections with horseradish peroxidase: John Wiley & Sons.
61. Honig MG, Hume RI (1989) DiI and diO: versatile fluorescent dyes for neuronal labelling and pathway tracing. Trends Neurosci 12: 333-335, 340-331.
62. Fabian RH, Coulter JD (1985) Transneuronal transport of lectins. Brain Res 344: 41-48.
63. Ericson H, Blomqvist A (1988) Tracing of neuronal connections with cholera toxin subunit B: light and electron microscopic immunohistochemistry using monoclonal antibodies. J Neurosci Methods 24: 225-235.
64. Schwab ME, Thoenen H (1976) Electron microscopic evidence for a transsynaptic migration of tetanus toxin in spinal cord motoneurons: an autoradiographic and morphometric study. Brain Res 105: 213-227.
65. Nance DM, Burns J (1990) Fluorescent dextrans as sensitive anterograde neuroanatomical tracers: applications and pitfalls. Brain Res Bull 25: 139-145.
66. Schmued L, Kyriakidis K, Heimer L (1990) In vivo anterograde and retrograde axonal transport of the fluorescent rhodamine-dextran-amine, Fluoro-Ruby, within the CNS. Brain Res 526: 127-134.
67. King MA, Louis PM, Hunter BE, Walker DW (1989) Biocytin: a versatile anterograde neuroanatomical tract-tracing alternative. Brain Res 497: 361-367.
68. Katz LC, Burkhalter A, Dreyer WJ (1984) Fluorescent latex microspheres as a retrograde neuronal marker for in vivo and in vitro studies of visual cortex. Nature 310: 498-500.
69. Smith BN, Banfield BW, Smeraski CA, Wilcox CL, Dudek FE, et al. (2000) Pseudorabies virus expressing enhanced green fluorescent protein: A tool for in vitro electrophysiological analysis of transsynaptically labeled neurons in identified central nervous system circuits. Proc Natl Acad Sci U S A 97: 9264-9269.
70. Banfield BW, Kaufman JD, Randall JA, Pickard GE (2003) Development of pseudorabies virus strains expressing red fluorescent proteins: new tools for multisynaptic labeling applications. J Virol 77: 10106-10112.
71. Standish A, Enquist LW, Escardo JA, Schwaber JS (1995) Central neuronal circuit innervating the rat heart defined by transneuronal transport of pseudorabies virus. J Neurosci 15: 1998-2012.
72. Zemanick MC, Strick PL, Dix RD (1991) Direction of transneuronal transport of herpes simplex virus 1 in the primate motor system is strain-dependent. Proc Natl Acad Sci U S A 88: 8048-8051.
