# Supplementary material for: A Proposal for a Coordinated Effort for the Determination of Brainwide Neuroanatomical Connectivity in Model Organisms at a Mesoscopic Scale
Source: PLoS Comput Biol. 2009 Mar 27;5(3):e1000334. doi: 10.1371/journal.pcbi.1000334 (PMC2655718; doi:10.1371/journal.pcbi.1000334)
Supplement: Text S2 — Example workflow, informatics requirements, timeline, and cost estimates for mouse connectivity project. Here we describe in greater detail a possible experimental pipeline and data analysis workflow for a systematic study of mesoscale mouse brain connectivity using neuroanatomical tracers. (1.43 MB PDF) [file pcbi.1000334.s002.pdf]

Text S2: Example workflow, informatics requirements, timeline, and cost estimates for mouse connectivity project

The chief proposal that we discuss in the main article is to systematically inject conventional neuronal tracers and/or viral vectors delivering fluorescent proteins into targeted brain regions in living, young adult mice, age and weight-matched to an existing stereotaxic atlas. Here we describe in greater detail a possible experimental workflow and pipeline using conventional tracers, the details of which are only meant to be illustrative (see Figure 1). A similar workflow using the same or very similar experimental apparatus may be developed for injected viral vectors, with appropriate modifications to the various processing stages. We have called for designed redundancy in the experimental program, resulting in multiple data points per injection region; such redundancy can also, for example, allow the use of both conventional and virus-based tracers at each brain site. The full details of the experimental protocols for such a project will need to be determined through pilot experiments and further consultations with experts.

Experimental procedures

Protocol for injections of neuronal tracers: Mice will be anesthetized and surgically fixed to a stereotaxic table, and a tracer injection guided by motorized micromanipulator to position the micropipette at a pre-determined location. Injection coordinates will be determined *a priori* for the full experimental program in order to approximately sample the entire gray matter. This process will require computational efforts before the experimental program begins to determine the optimal distribution of injection sites, factoring in known anatomy. A high-resolution tracer with anterograde preference (BDA in this example, or PHA-L with slight modifications) and tracer with retrograde preference (CTB) will be co-injected from the micropipette (10-20 μm diameter) using iontophoresis, roughly according to previous protocols [e.g. Ref. 1], or using microsyringes (e.g. Hamilton) and pressure injection. The injection process must be carefully controlled (electromechanically if feasible) to ensure regular injection volumes and locations, and injections will be analyzed *post hoc* to remove unsuccessful experiments. Injected mice will rest in a cage during a survival period of approximately 7-10 days to allow for sufficient tracer transport; standardized values for this and other experimental parameters can be determined during pilot studies. The animal will then be deeply anesthetized and perfused transcardially using a 4% paraformaldehyde solution, and the brain removed. The brain will be rapidly frozen and sectioned using a cryostat to cut coronal sections of ~30 μm thickness. Section plane orientation must be standardized and reproducible across experiments and pipelines, thus necessitating the use of customized brain blockers or other apparatus to assure reproducibility. Further, during the sectioning process, block face digital photographs of each section should be acquired prior to cutting [e.g. 2,3], providing an aligned reference for automated serial reconstruction algorithms (see below).

Tissue processing: The approximately 500 resulting sections must then be assembled into matched series of sections and mounted onto slides. A subset of sections should be stained for cellular and/or chemical microarchitecture, with the remaining slides processed for detection of the tracer substances. Sections stained to reveal cell bodies with thionin or NeuN antibody (for neuron-specific labeling) can be used *post hoc* to help localize tracer injection sites and labeled cellular processes (from adjacent sections) relative to the cytoarchitecture. These images will also have possible utility in enabling high-resolution registration of individual brains to a

common template space. Additional sections can be stained for myelin using Luxol fast blue or hematoxylin or processed for other attributes such as types of neurotransmitters. Because the primary objective of the project must remain connectivity tracing, it is advisable that a large fraction of available sections (e.g. 1 out of 2-5) be used for tracer label detection, and that the remaining slides be rationed among other processes deemed valuable to the project. The number of additional histological procedures should thus be restricted to ensure that data from different modalities are within sufficient proximity to preserve the resolution of the overall data set.

Histochemical processing: Detection of two unique labels from the anterograde and retrograde tracer in a multiple injection protocol will proceed using a standardized procedure resulting in two distinct and permanent reaction products. BDA detection, for example, only requires incubation in avidin-HRP for approximately 90 minutes, followed by several washes in phosphate buffer (PB), then followed by incubation in DAB-Ni (diaminobenzidine containing low concentrations of nickel ammonium sulfate) for 5-10 minutes, resulting in a blue-black reaction product. CTB detection requires a longer but still systematic series of incubations, first in rabbit anti-cholera toxin antiserum for 24 hours, followed by goat anti-rabbit IgG for 60 minutes, rabbit-PAP for 60 minutes, and finally standard DAB, yielding a red-brown reaction product [see Ref. 4]. The above procedures typically require much manual skill and parameters differ from laboratory to laboratory, but in the proposed large-scale effort, they will be standardized accordingly. Additionally, automation will be added to the process where technically and economically feasible to reduce human bias and error. The Allen Brain Atlas (ABA) project, for example, demonstrated the feasibility of incorporating automation into a histological pipeline for research by using commercially-available robotics for liquid handling in their *in situ* hybridization protocol [Ref. 5; see Supplemental Methods 1]. Multiple vendors supply equipment for automated administration of staining and immunohistochemistry protocols and for coverslipping. Such technologies can reduce human bias and error within and across participating laboratories.

Microscopy: For the imaging stage, equipment is now available for semi-automated scanning and digitization in compressed image format of the labeled sections at sub-micron resolution using fluorescence or bright field microscopy (see also discussion of experimental methods in additional supplementary materials). Depending on section thickness and performance of microscope technologies and image processing algorithms, it may be beneficial to acquire a multiple-image stack for each section, imaged at different focal depths in the z-plane, to enable better segmentation of cell bodies and axon fibers. It is important that each site involved in the project have access to the same microscopy technologies to ensure consistency in the raw image data, and unbiased performance of signal extraction algorithms across locations. For each experiment, the complete set of treated slides (covering the entire brain) will be scanned. The digitized images will then be transferred into a distributed data processing pipeline for automated analysis of the experimental results (see below), and rapidly submitted to a public data repository that will serve not only the *results* from this project but also the *raw* image data. The physical slides can be stored locally at the site performing the experiment.

Automated data analysis

As the primary data elicited from a high-throughput tract tracing program are images, the major informatics challenge will be to set up an automated pipeline for processing the many image

sections. The primary image processing tasks are 1) automated digitization and storage of microscopy images; 2) 3D reconstruction of anatomy from serial sections; 3) registration of different test brains to a common coordinate space; 4) detection and quantification of labeled portions of the images, and quantitative assessment of the strength and/or density of labeling; and 5) characterization of normal individual variation. The first four challenges are reasonably similar to those that have been successfully met by the ABA project [6], and these efforts should serve as a guide. The 5th task is an important addition to the current project, and will additionally set the stage for the study of abnormal variation in *connectivity phenotypes* using the same procedures to study mouse disease models.

Using acquired block-face digital images as a reference, the full set of sections (including those stained for Nissl, etc.) for an individual brain can be reconstructed into a 3D volume [7] with a global coordinate system. A variety of image warping methods [8,9] can be employed to register the image data for each digitized section with the corresponding block face image, matching image contours or landmarks, or by, for example, maximizing mutual information between the images [10]. This stage will need to account for alterations in the tissue shape due to histological processing, and for tissue shrinkage. After reconstruction, individual brains should be registered to a common atlas coordinate space to enable common presentation of results and analysis across individual experiments. This necessitates matching of the global shape of the individual volume with the atlas volume, which may be augmented by local image deformations to approximately match individual brain regions. The resulting deformation fields can be used to bring signals extracted from the individual section data (e.g. the axon segments and cell bodies) into the common atlas space.

A critical module needed for the proposed experimental program will contain algorithms to automatically detect and quantify labeled axonal fibers and cell bodies. Currently the usual procedure for charting labeled cells uses either a camera lucida or semi-automated image-combining computerized microscopy (e.g. the NeuroLucida system [11]), which allows the experimenter to digitally record coordinate-based data in the same space relative to an image under the microscope. With the incorporation of the stage of high-quality, high-resolution imaging of all primary data, it is quite feasible to automate the detection of the injection site and extent, as well as labeled cell bodies and fibers *throughout the entire brain* using post-hoc image analysis. The ABA employed such automated image processing steps to detect cell bodies labeled through *in situ* hybridization [6], and progress has been made toward automatically detecting and measuring labeled axonal fibers and plexuses [12,13,14]. These challenges amount to segmentation problems in image processing, and a wide variety of methods can be brought to bear that are likely to prove reliable in the controlled scenarios we have proposed. The output of these signal detection algorithms should include a quantitative evaluation of the location and extent of the injection site as determined by densely labeled cell bodies centered on the prescribed stereotaxic coordinates, and quantitative estimates of the fiber density and number and locations of labeled cells at projection target and source sites, respectively. Sites with very low density labeling may be ignored, as these could be due to fibers of passage or transport in the non-preferred direction resulting in labeling of axon collaterals.

Laboratory information management system and data storage

The proposed high-volume, multi-site experimental workflow demands an integrated, computerized laboratory information management system (LIMS) that seamlessly organizes and manages the sets of discrete tasks, protocols, and materials across laboratories. This is a critical component, particularly for projects distributed across sites, which is likely to require custom development to meet project needs. Accordingly, LIMS development and implementation will be a significant project expense, included in the coarse budget estimate in additional supplementary information. To the extent possible, the LIMS will automate planning and quality control, track materials (e.g. using bar codes), record various experimental parameters, and issue work lists to technicians and pieces of equipment at the different project sites.

The system should also link all experimental metadata with the digitized image data, which will necessitate massive, redundant data storage capabilities and computational power over a fast, distributed network. An estimate of raw image data alone indicates storage requirements on the order of a petabyte; only recently has the storage of such voluminous data become feasible as hard drive density has increased exponentially through time according to Kryder's Law [15] alongside a concomitant decrease in storage costs. The overall processing pipeline must be able to coordinate operations across different software tools and assure quality assurance through the employment of various test cases. If the data processing pipeline is implemented across sites, additional efforts will be required to coordinate the analyses. Systems to integrate data and processing pipelines across laboratories are currently being implemented, for instance, in the domain of human neuroimaging [16].

Representation and dissemination of information

Neuroanatomical data model: A major challenge is to develop an appropriate schema to represent the results of the injection experiments and associated metadata. In previous databases for connectivity information based on literature curation (CoCoMac, BAMS), the underlying model of anatomy is discrete; that is, each "connection" is associated with a pair of brain regions that are defined in the continuous brain space. Through systematic injections, and by preserving and storing original microscopy data, we can avoid such discretization and represent the results in analog form. Spatial databases [17] as used in geographical information systems provide many of the necessary tools once the underlying data model (e.g. coordinate system) has been established. Anatomical parcellations based on different atlases may then be probabilistically registered to this coordinate space to enable the representation of the full connectivity data in the form of connectivity graphs or matrices, with "nodes" defined by the particular parcellation. Furthermore, representation in the continuous space allows for a post-hoc analysis that *solves for* the partitioning of brain space that best follows the connectivity patterns observed in the data.

It is also of note that the results collected will span different levels of resolution in brain organization, from sub-neuronal compartments, neuronal compartments, neurons, groups of neurons, brain regions, to other high-level anatomical entities. The database schema should support queries designed to ask questions about various levels of organization, and should be extensible to allow incorporation of additional anatomical information, for example about cyto- or chemo-architecture or about genetic profiles of spatially localized brain areas or cell populations. The schema must support rich metadata of various forms; for the new experimental data generated within the project, the recording of all experimental parameters, dates and times,

and comments from the experimenters must be explicitly built into the experimental protocols and should be as simple and automated as possible.

Interfaces for data access and visualization: Finally, a web-based portal should provide complete access to the data set and processed results. The "raw" data slides should be made available for fast retrieval as multi-resolution zoomable images [e.g. Ref. 18]. The primary interface for selecting and visualizing results should be a highly interactive, customizable 3D anatomical brain model (which should support rotate, pan, zoom, drag, highlight, and so on) in the spirit of tools like Google Earth [see Ref. 19]. Efficient visualization of the results is of high importance, and failures in this regard have likely been one reason for the slow adoption of database technologies in neuroscience. User interfaces will also need to support user retrieval of primary data and interoperability with other databases through Web Services and via the Semantic Web [20], leveraging emerging data protocols like XML (extensible markup language), RDF (resource description framework), and OWL Web Ontology Language.

User groups and future efforts: We anticipate this project will be useful to nearly all neuroscience researchers, but will be of particular interest to several groups. Because of the biomedical motivation for this project, specific interfaces to the data should be targeted toward clinical researchers and clinical trainees. Furthermore the informatics portion of the project should make every effort to integrate existing knowledge regarding the anatomical bases for neurological and neuropsychiatric disorders in animal models and through homology with humans into the knowledge base. This may best be achieved through the establishment of collaborations with medical researchers. Additional prominent user groups are expected to include theoreticians working on systems-level brain models, evolutionary neurobiologists, and researchers in functional imaging.

An important element of this coordinated collaborative effort will be to promote the use of the data set and results within the community. Furthermore, it is expected that such a large-scale project can reinvigorate and nucleate a modern neuroanatomy community. In conjunction with the suggestions outlined here, we propose the development of a new annual neuroanatomy summer course centering around these methodologies and results to help achieve such goals.

Cost estimates and timeline

This workflow essentially describes the events that would take place at a single experimental "pipeline," of which there may be several, located at multiple sites devoted to the project. We have made coarse estimates of the equipment and personnel required to implement such experimental pipelines to carry out the proposed mouse connectivity project. The estimate is based on 5 replicates of the experimental pipeline. The major pieces of equipment required at each replicate are:

1. Mouse stereotaxic frame with motorized micromanipulator and electrode holder
2. Electrode puller
3. Current source for iontophoresis
4. Basic surgical equipment
5. Refrigeration equipment
6. Cryostat

7. Equipment for staining, immunohistochemistry, and liquids processing
8. Equipment for slide coverslipping
9. Virtual microscopy / slide scanning system

The total capital cost for one experimental "pipeline" is estimated at ~$500k USD. Assuming 5 such pipelines in distributed laboratories the estimated capital cost for experimental equipment is on the order of $2.5M. Each experiment furthermore requires certain supplies including the tracer substances and other reagents, slides and cover slips, pipettes and the mice. Here we estimate the cost for such supplies to be approximately $200 per experiment. After a startup delay, each pipeline should conservatively be capable of processing 2 experiments per day[†], or approximately 500 per year, for a recurring experimental supply cost of approximately $100k per year, per pipeline.

We assume the following personnel associated with each pipeline: 1 research assistant professor, 1 animal technician, 1 surgery / injection technician, and 1 histology / imaging technician. At an average cost of $100k per year for each of 4 staff members x 5 pipelines, the yearly total personnel cost for the experimental portion of the proposed program is estimated to be $2M. Additional costs will be accrued for informatics equipment and personnel, detailed below. We also summarize total expected costs and the anticipated timeline in a final section below.

Informatics Cost Estimates: The cost of data storage is estimated at $2 USD per gigabyte. We calculate that ~50 GB of storage is sufficient for the redundant storage of raw and processed images and metadata for each experiment, equal to $100 per experiment. Computational and server nodes plus network backbone will be a significant capital cost, approximated to be on the order of $1M plus approximately $500k for laboratory information management system (LIMS). To assess personnel costs, we assume the informatics portion of the project, which will be largely centralized, will be led by 1 research assistant professor, and will also include 2 IT technicians / programmers, and 2 postdoctoral associates. At an average of $100k annually, the IT personnel costs are on the order of $500k per year.

Overall costs and timeline: A summary of the proposed 5-year timeline for the mouse project is given in Figure 2. Total cost calculations are based on assumptions that the full project will require 10,000 injection experiments; given the Bota et al. (2003) estimate of 500-1000 unique cell groups (in rat), this number should be sufficient to supply multiple data points per region, and to allow for unavoidable errors. It is expected that the first project year will involve technical setup of experimental and IT infrastructure, and the experiments themselves will begin in Year 2. Thus, we estimate the need for 2,500 experiments per year, or about 10 per day. This could likely be carried out by 5 experimental work groups ("pipelines") at distributed laboratories with 4 staff members per group, as described above. Data storage costs (see above) were estimated at $100 per experiment, for a total of $1M for all experimental data. Adding capital costs for experimental equipment, virtual microscopy, and IT infrastructure plus salary for personnel (the largest expense in the project), we estimate the total cost for carrying out the proposed mouse connectivity project to be approximately $19.5M.

[†] Note that while the course of an individual experiment is longer than one day, we estimate that 2 animals can be taken through a particular stage of the experiment (e.g. the surgery and injection) at each pipeline in a single standard work day. This number establishes the throughput of the pipeline.

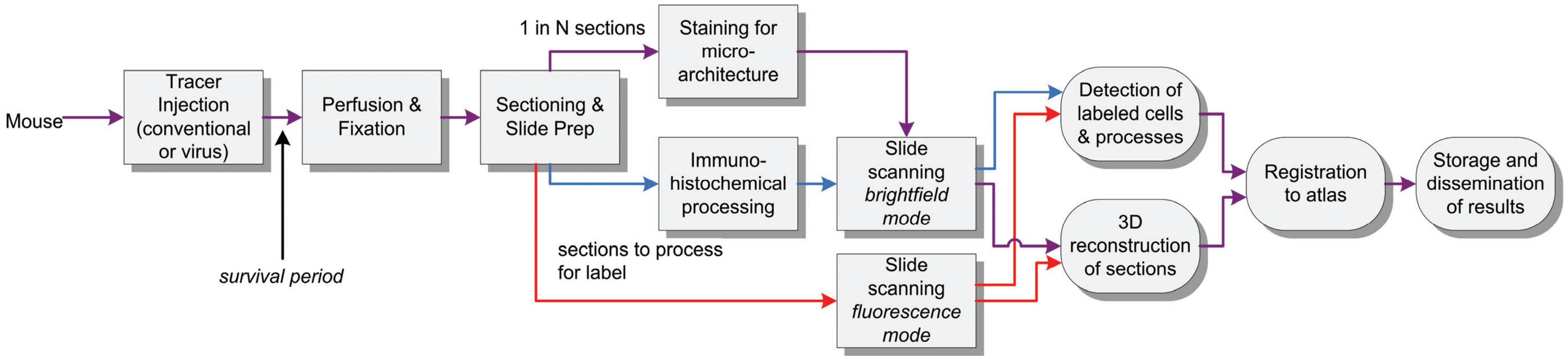

Figure 1: Basic workflow for a single connectivity experiment in the proposal using injections of either conventional tracers or viral vectors. Square boxes indicate stages requiring primarily experimental apparatus; rounded boxes are implemented primarily in software. Arrows represent the flow of materials (tissue, images) through the pipeline. Red arrows indicate the flow for experiments using viral tracers (and fluorescent microscopy), blue arrows indicate flow for conventional tracers, and purple arrows for flow of materials in either method.

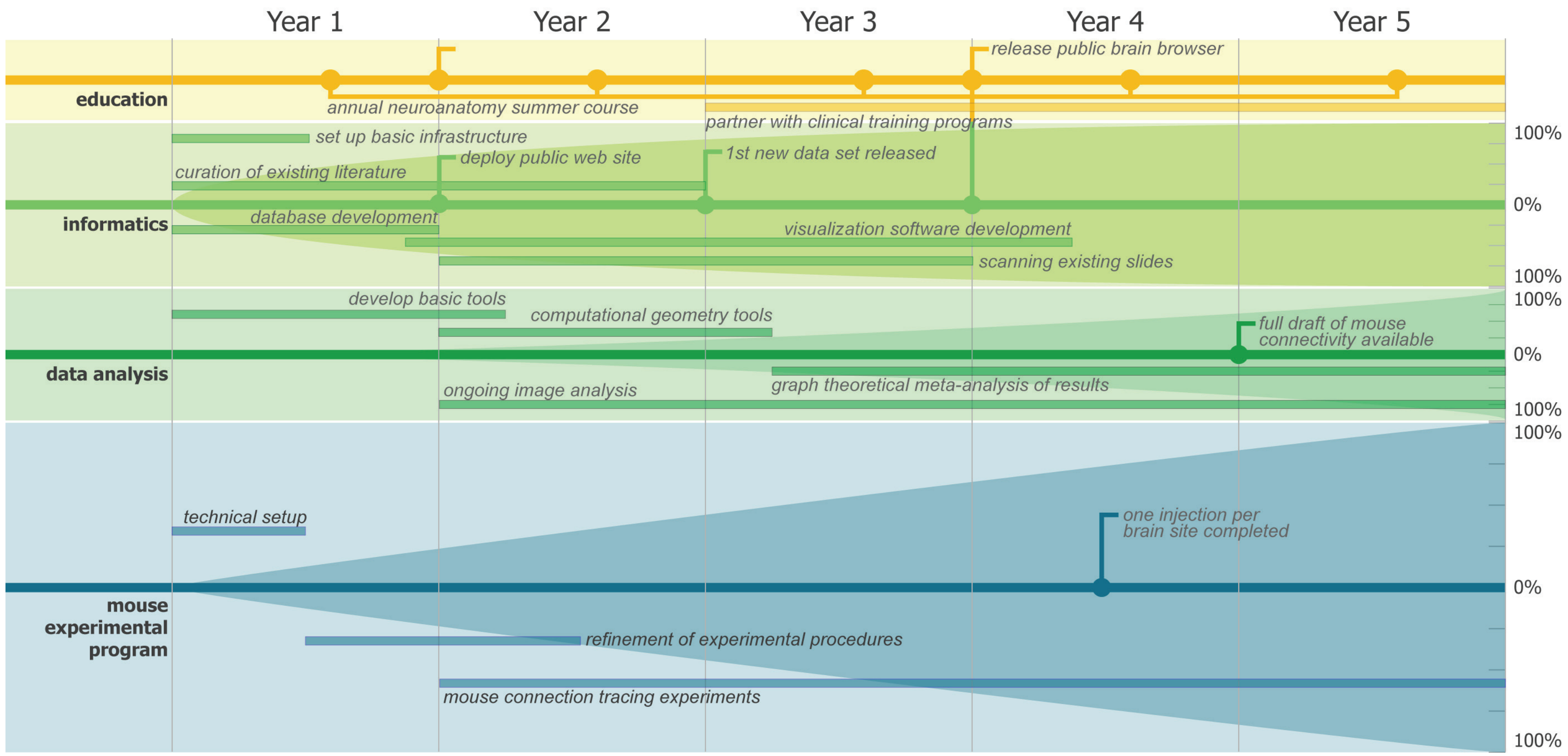

Figure 1: Proposed project timeline: a schematic illustration of how the proposed project would unfold in time. The project is divided into several components as depicted by the colored “bands” that run horizontally (through time). The relative thickness of each band indicates the relative distribution of project resources to that component. Events or projected milestones appear as circles on each axis, and subprojects appear as horizontal bars within each band. Finally, a set of curves in the background of each band represents the expected degree of completion of that portion of the project throughout the proposed five year span.

References

1. Coolen LM, Wood RI (1998) Bidirectional connections of the medial amygdaloid nucleus in the Syrian hamster brain: simultaneous anterograde and retrograde tract tracing. J Comp Neurol 399: 189-209.
2. MacKenzie-Graham A, Lee EF, Dinov ID, Bota M, Shattuck DW, et al. (2004) A multimodal, multidimensional atlas of the C57BL/6J mouse brain. J Anat 204: 93-102.
3. Toga AW, Banerjee PK (1993) Registration revisited. J Neurosci Methods 48: 1-13.
4. Coolen LM, Jansen HT, Goodman RL, Wood RI, Lehman MN (1999) A new method for simultaneous demonstration of anterograde and retrograde connections in the brain: co-injections of biotinylated dextran amine and the beta subunit of cholera toxin. J Neurosci Methods 91: 1-8.
5. Lein ES, Hawrylycz MJ, Ao N, Ayres M, Bensinger A, et al. (2007) Genome-wide atlas of gene expression in the adult mouse brain. Nature 445: 168-176.
6. Ng L, Pathak SD, Kuan C, Lau C, Dong H, et al. (2007) Neuroinformatics for genome-wide 3D gene expression mapping in the mouse brain. IEEE-ACM Transactions on Computational Biology and Bioinformatics 4: 382-393.
7. Bjaalie JG, Leergaard TB (2006) Three-dimensional computerized reconstruction from serial sections: cells populations, regions, and whole brain. In: Zaborszky L, Wouterlood FG, Lanciego JL, editors. Neuroanatomical Tract Tracing: Molecules, Neurons, & Systems. New York: Springer. pp. 530–565.
8. Hill DL, Batchelor PG, Holden M, Hawkes DJ (2001) Medical image registration. Phys Med Biol 46: R1-45.
9. Toga AW, Thompson P (1999) An introduction to brain warping. Brain Warping: 1Â–26.
10. Pluim JP, Maintz JB, Viergever MA (2003) Mutual-information-based registration of medical images: a survey. IEEE Trans Med Imaging 22: 986-1004.
11. Glaser JR, Glaser EM (1990) Neuron imaging with Neurolucida--a PC-based system for image combining microscopy. Comput Med Imaging Graph 14: 307-317.
12. Broser PJ, Erdogan S, Grinevich V, Osten P, Sakmann B, et al. (2008) Automated axon length quantification for populations of labelled neurons. J Neurosci Methods 169: 43-54.
13. Lillehaug S, Oyan D, Leergaard TB, Bjaalie JG (2002) Comparison of semi-automatic and automatic data acquisition methods for studying three-dimensional distributions of large neuronal populations and axonal plexuses. Network 13: 343-356.
14. Schwarz C, Mock M (2001) Spatial arrangement of cerebro-pontine terminals. J Comp Neurol 435: 418-432.
15. Walter C (2005) Kryder's law. Scientific American 293: 32-33.
16. Keator DB, Grethe JS, Marcus D, Ozyurt B, Gadde S, et al. (2008) A national human neuroimaging collaboratory enabled by the Biomedical Informatics Research Network (BIRN). IEEE Trans Inf Technol Biomed 12: 162-172.
17. Güting RH (1994) An introduction to spatial database systems. The VLDB Journal The International Journal on Very Large Data Bases 3: 357-399.
18. Mikula S, Trotts I, Stone JM, Jones EG (2007) Internet-enabled high-resolution brain mapping and virtual microscopy. Neuroimage 35: 9-15.
19. Butler D (2006) Virtual globes: the web-wide world. Nature 439: 776-778.
20. Berners-Lee T, Hendler J, Lassila O (2001) The Semantic Web - A new form of Web content that is meaningful to computers will unleash a revolution of new possibilities. Scientific American 284: 34-43.
