# Supplementary material for: A Proposal for a Coordinated Effort for the Determination of Brainwide Neuroanatomical Connectivity in Model Organisms at a Mesoscopic Scale
Source: PLoS Comput Biol. 2009 Mar 27;5(3):e1000334. doi: 10.1371/journal.pcbi.1000334 (PMC2655718; doi:10.1371/journal.pcbi.1000334)
Supplement: Text S3 — Brief proposal for primate connectivity project. We describe an approach to better understand connectivity in the macaque brain that includes collating and digitizing existing materials as well as implementing specifically targeted experiments with standard protocols. (0.15 MB PDF) [file pcbi.1000334.s003.pdf]

Text S3: Brief proposal for primate connectivity project

As discussed in the primary article, a high-throughput investigation on the same scale as that proposed for mouse is not currently feasible in primates. While a smaller number of specifically targeted experiments in Macaque should provide highly valuable information, much effort should also be put into collating, representing, and making available the results of previous studies. Specifically this should include digitizing legacy slides, where possible, and preserving the physical slide libraries of researchers who are leaving the field. The completion of new experimental studies should then be used to "fill in" key gaps of existing knowledge or to target circuits of special importance. Such experiments should again follow standardized protocols to the extent possible (though these may differ considerably from those used in mouse) to avoid the problem of heterogeneous results that are difficult to integrate in a single global framework.

Literature curation and capture of existing raw data: The value of any individual connectivity study that has previously been performed in non-human primates is elevated relative to those in rodent due to the increased cost and difficulty of experimental work in primates. The CoCoMac database[1,2] currently serves an important role by gathering, through manual curation of the literature, many previous findings in macaque. While this effort should be both applauded and expanded, extraction of knowledge from articles has considerable limitations, primarily due to a lack of consistency in how results have been reported. Furthermore, textual descriptions of connections in the literature can only be considered to be an *interpretation* of the underlying data. The lack of availability of the primary source materials, beyond published images which provide only a partial view, leads to considerable uncertainty in reconciling different results. Compilations of multiple experimental results from the same laboratory using similar techniques (e.g. tritiated amino acids, HRP, BDA and other dextrans, and fluorescent dyes) provide substantial unified views of the neural connections of several cortical and subcortical structures in primates (for reviews of original papers see Refs. [3,4,5,6]). Still, a significant effort must be made to not only curate the existing literature, but to probabilistically map these results into a common 3D template space. This will help to provide an understanding of the "missing data," and will allow previous results to be viewed in the same context with targeted investigations going forward.

The collation and curation of previous results could be considerably augmented if a portion of the primary image data on which reported results were based was made available in digital form[†]. A first step in the primate portion of the project, thus, should be a concerted effort to digitize available materials in high quality using the now available technologies for virtual microscopy (see discussion of experimental methods in Text S1). Practically this could be carried out either by funding the purchase of such equipment for multiple labs that have produced large quantities of primary data, or in a centralized model in which samples are transported to a common facility for digitization and then returned to the experimentalists. Such an effort would yield a more comprehensive picture of the state of current knowledge by facilitating a more accurate *geographic* interpretation of the data (see also discussion of informatics in Text S2), rather than one based on subjective textual description. This would require substantial computational effort to map the scanned images to a common geometric

---

[†] It is unfortunately true that a considerable portion of the primary data, particularly involving fluorescent tracers, will no longer be visible due to degradation through time.

template, but such efforts could be refined over time once the primary data became digitally available. In addition to the digital preservation of legacy slides, a central library should be created to preserve the physical slides and to allow access to future researchers. This is particularly important as many classically trained neuroanatomists will be leaving the field in upcoming years, and their combined collections are a valuable resource that must not be discarded. For curated and digitized legacy data, heterogeneous metadata must capture as much information about the source data as is available. The database system should also allow the original experimentalists to annotate the legacy data by, for example, allowing "layers" of metadata (e.g. anatomical annotations) to be stored with spatial reference to the image data.

Experimental program: As stated above, a concerted experimental effort for macaque should be focused where current knowledge is determined to be most lacking, while also considering the potential scientific and/or biomedical impact of the new data. For macaque studies, a multiple-labeling protocol is again proposed, though we will not describe this in detail. In principle, the conventional tracer methods described for the mouse can be similarly applied in macaque, though a few important modifications should be made.

First, a high-quality T1-weighted anatomical magnetic resonance imaging (MRI) scan (with 1 cubic millimeter or less voxel size) should be obtained for each experimental subject. This added preliminary stage will greatly assist in the localization of injections, in anatomical reconstruction, and in registration of individual brains to a common template. Injection coordinates specific to each animal can be derived from analysis of the MR volume. A second imaging data set based on diffusion spectrum imaging (DSI)[7] would have added scientific benefits if acquired in some animals, as comparison of the results of tracer injections could be compared directly with the imaging tractography in order to validate the method [see Ref. 8]. The addition of the imaging stage(s) is valuable and can be completed in a short time period, but will require the availability of imaging facilities and will add substantial cost.

The tracer protocols require additional updates and must be standardized for the macaque. The iontophoretic injection method is not desirable for this level of investigation in the larger primate brain and should be replaced with controlled air pressure injections. Additionally, the tracer protocol can be expanded to include several additional tracers (see, e.g. Ref. [9]) and/or multiple injection sites[10,11]. A further substantive difference will be in the post-injection survival period, which will be extended to approximately 14-18 days for the macaque. Viral methods that label multi-synaptic pathways may provide additional information in some systems, though they are more expensive and may require special laboratory equipment and expertise. These methods are particularly viable if experiments can be implemented in existing laboratories that currently use these techniques.

Current progress being made on primate brain connectivity can be seen at BrainMaps.org[12], where sub-micron image data from recent tracer experiments using CTB, injected into various cortical areas, are directly accessible online. These ongoing efforts will result in a multi-terabyte online database of virtual slides that will enable high-resolution mapping of connectivity patterns in the primate and will drive subsequent neuroinformatics and data mining initiatives.

References:

1. Kotter R (2004) Online retrieval, processing, and visualization of primate connectivity data from the CoCoMac database. Neuroinformatics 2: 127-144.
2. Stephan KE, Kamper L, Bozkurt A, Burns GA, Young MP, et al. (2001) Advanced database methodology for the Collation of Connectivity data on the Macaque brain (CoCoMac). Philos Trans R Soc Lond B Biol Sci 356: 1159-1186.
3. Barbas H, Ghashghaei HT, Rempel-Clower NL, Xiao D (2002) Anatomic basis of functional specialization in prefrontal cortices in primates. In: Grafman J, editor. Handbook of Neuropsychology, Second Edition. Amsterdam: Elsevier Science. pp. 1-27.
4. Haber SN (2003) The primate basal ganglia: parallel and integrative networks. J Chem Neuroanat 26: 317-330.
5. Jones EG (1998) A new view of specific and nonspecific thalamocortical connections. Adv Neurol 77: 49-71; discussion 72-43.
6. Schmahmann JD, Pandya DN (2006) Fiber pathways of the brain. New York: Oxford University Press.
7. Wedeen VJ, Hagmann P, Tseng WY, Reese TG, Weisskoff RM (2005) Mapping complex tissue architecture with diffusion spectrum magnetic resonance imaging. Magn Reson Med 54: 1377-1386.
8. Schmahmann JD, Pandya DN, Wang R, Dai G, D'Arceuil HE, et al. (2007) Association fibre pathways of the brain: parallel observations from diffusion spectrum imaging and autoradiography. Brain 130: 630-653.
9. Saleem KS, Kondo H, Price JL (2008) Complementary circuits connecting the orbital and medial prefrontal networks with the temporal, insular, and opercular cortex in the macaque monkey. J Comp Neurol 506: 659-693.
10. Lanciego JL, Luquin MR, Guillen J, Gimenez-Amaya JM (1998) Multiple neuroanatomical tracing in primates. Brain Res Brain Res Protoc 2: 323-332.
11. Zikopoulos B, Barbas H (2007) Parallel driving and modulatory pathways link the prefrontal cortex and thalamus. PLoS ONE 2: e848.
12. Mikula S, Trotts I, Stone JM, Jones EG (2007) Internet-enabled high-resolution brain mapping and virtual microscopy. Neuroimage 35: 9-15.
